# Synthesis, Characterization and Biomedical Application of Silver Nanoparticles

**DOI:** 10.3390/ma15020427

**Published:** 2022-01-06

**Authors:** Ashwini Naganthran, Gayathiri Verasoundarapandian, Farah Eryssa Khalid, Mas Jaffri Masarudin, Azham Zulkharnain, Norazah Mohammad Nawawi, Murni Karim, Che Azurahanim Che Abdullah, Siti Aqlima Ahmad

**Affiliations:** 1 Department of Biochemistry, Faculty of Biotechnology and Biomolecular Sciences, Universiti Putra Malaysia, Serdang 43400, Selangor, Malaysia; ashwininaganthran@gmail.com (A.N.); gayathiri1802@gmail.com (G.V.); faraheryssa@gmail.com (F.E.K.); 2 Department of Cell and Molecular Biology, Faculty of Biotechnology and Biomolecular Sciences, Universiti Putra Malaysia, Serdang 43400, Selangor, Malaysia; masjaffri@upm.edu.my; 3 Department of Bioscience and Engineering, Shibaura Institute of Technology, College of Systems Engineering and Science, 307 Fukasaku, Saitama 337-8570, Japan; azham@shibaura-it.ac.jp; 4 Institute of Bio-IT Selangor, Universiti Selangor, Jalan Zirkon A7/A, Seksyen 7, Shah Alam 40000, Selangor, Malaysia; norazah@unisel.edu.my; 5 Centre for Foundation and General Studies, Universiti Selangor, Jalan Timur Tambahan, Bestari Jaya 45600, Selangor, Malaysia; 6 Department of Aquaculture, Faculty of Agriculture, Universiti Putra Malaysia, Serdang 43400, Selangor, Malaysia; murnimarlina@upm.edu.my; 7 Laboratory of Sustainable Aquaculture, International Institute of Aquaculture and Aquatic Sciences, Universiti Putra Malaysia, Port Dickson 71050, Negeri Sembilan, Malaysia; 8 Department of Physics, Faculty of Science, Universiti Putra Malaysia, Serdang 43400, Selangor, Malaysia; azurahanim@upm.edu.my; 9 Material Synthesis and Characterization Laboratory, Institute of Advanced Technology, Universiti Putra Malaysia, Serdang 43400, Selangor, Malaysia; 10 Laboratory of Bioresource Management, Institute of Tropical Forestry and Forest Products (INTROP), Universiti Putra Malaysia, Serdang 43400, Selangor, Malaysia

**Keywords:** physical, chemical, biological, AgNPs, design and synthesis methods of silver nanoparticles, biomedical properties of AgNPs, biomedical applications, action mechanism

## Abstract

Silver nanoparticles (AgNPs) have been employed in various fields of biotechnology due to their proven properties as an antibacterial, antiviral and antifungal agent. AgNPs are generally synthesized through chemical, physical and biological approaches involving a myriad of methods. As each approach confers unique advantages and challenges, a trends analysis of literature for the AgNPs synthesis using different types of synthesis were also reviewed through a bibliometric approach. A sum of 10,278 publications were analyzed on the annual numbers of publication relating to AgNPs and biological, chemical or physical synthesis from 2010 to 2020 using Microsoft Excel applied to the Scopus publication database. Furthermore, another bibliometric clustering and mapping software were used to study the occurrences of author keywords on the biomedical applications of biosynthesized AgNPs and a total collection of 224 documents were found, sourced from articles, reviews, book chapters, conference papers and reviews. AgNPs provides an excellent, dependable, and effective solution for seven major concerns: as antibacterial, antiviral, anticancer, bone healing, bone cement, dental applications and wound healing. In recent years, AgNPs have been employed in biomedical sector due to their antibacterial, antiviral and anticancer properties. This review discussed on the types of synthesis, how AgNPs are characterized and their applications in biomedical field.

## 1. Introduction

Nanotechnology is one of the fastest growing field and its application can be applied in various industry sectors. This technology helps to downsize bigger size materials with unique properties to atomic size and these are called nanoparticles (NPs). NPs are particles that ranges between 1 to 100 nm. These NPs are unique due to their small size, large surface area to volume ratio, high carrier capacity, high reactivity and easy variation of surface properties [1]. The distinctive properties of smaller-sized AgNPs also enable them to be applied in wide range of functions [2]. Currently, silver nanoparticles (AgNPs) have been widely used in agriculture, commercial, medical and industry applications [3]. AgNPs are used as an additive in vaccine adjuvant, anti-diabetic agent, wound and bone healing, biosensors and anticancer therapy in medical applications [4]. 

In agriculture, AgNPs are incorporated in nanopesticides and nanofertilizers [5]. These NPs are unique and have different surface to volume-ratio based on the type of synthesis. Smaller AgNPs has a larger surface to volume ratio, which release more silver cations, hence they are more effective as antimicrobial agents as compared to a bigger sized AgNPs [6]. Bigger AgNPs, which are more than 100 nm in size, have smaller surface to volume ratio and are usually used in drug delivery due to the quantity of drug delivered [7]. This size of the particles is essential for the transport through the certain membranes in the human body. Generally, nanoparticles have been produced through various preparations, including physical, chemical and biological routes. The physical and chemical synthesis of AgNPs is oftentimes hazardous and is less cost-effective [8,9] whilst biological synthesis, conversely, are able to produce AgNPs with a higher yield, solubility, stability and biocompatibility [9].

Studies utilizing AgNPs have been of great interest in recent years, with this furthered by the development of antibiotic resistance in many microbial pathogens over time [10]. Nanomaterials have increasingly become a staple in biomedicine, which lead to formation of nanobiotechnology [11]. The application of nanomaterials in medicine is still in research and development stage. Interestingly, silver (Ag) has been widely used in medical treatment and management with variety of diseases since ancient times. AgNPs also are one of the noble metallic NPs that have gained the most attention and exhibited the highest-level commercialization, which recorded 55.4% of the nanometals [12]. AgNPs and silver-based compounds are well known in this field due to their microbial killing potential [13,14]. Currently, AgNPs play roles primarily in non-conventional and enhanced biomedical applications such as wound dressing, drug delivery, tissue scaffoldings and proactive coating applications. In this regard, the AgNPs are considered to possess intrinsic features such as attractive chemical and physical functionality, non-toxic nature, wide spectrum of bactericidal properties, anticancer properties, and therapeutic abilities [15]. Hence, the applicability of AgNPs have increased in nanotechnology, biomedicine, and environment, thus there is a need for development of cost-effective method for the biosynthesized AgNPs [16].

## 2. General Synthesis Routes of AgNPs

**Figure 1:**
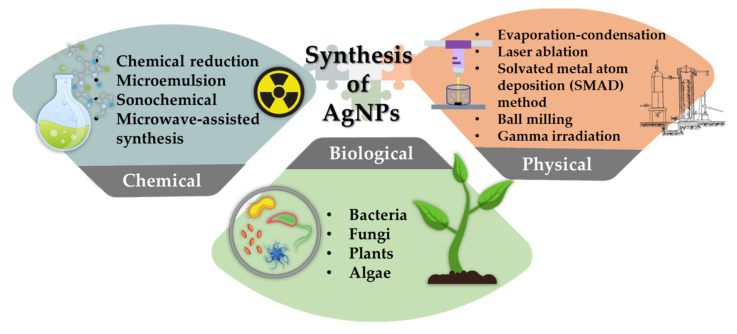
Synthesis of AgNPs.

The synthesis of AgNPs, like for most nanomaterials, is mainly divided into three different processes: chemical, physical and biological synthesis (Figure 1). Physical synthesis refers to synthesis of AgNPs from bulk materials to nanostructures using various physical forces [7]. Agglomeration is facilitated by the absence of capping or stabilizing agents. As a result, deterring it poses a significant challenge. Additionally, this synthesis requires external energy and sophisticated equipment.

Chemical and biological synthesis assembles single atoms and molecules into larger nanostructure to produce AgNPs. Chemical and biological synthesis also refers to synthesis of AgNPs by molecular components by nucleation and followed by growth. The chemical and biological methods can obtain AgNPs by reducing the precursor salt. Various shapes of AgNPs can be obtained quickly by chemical synthesis, but these AgNPs have limitations in medical application due to its toxicity. The synthesis involved usage of harsh chemical and may be harmful to the environment. Hence, to overcome this issue, the biological synthesis was introduced as an alternative method. It is reported that biopolymers used in AgNPs synthesis were fucoidan, chitosan, ascorbic acid, levan, cellulose, polyphenols and polypeptides [17,18,19,20]. Most of the biopolymers used in the synthesis of AgNPs play the dual role of reducing and stabilizing agent except the starch was used as a capping agent. Thin layer of biopolymers coat the surface of AgNPs, which acts as stabilizing and capping agent. The modified surface showed improved biocompatibility, intracellular uptake for drug delivery and longer stability [21].

Bibliometric analysis is defined as a method of statistical evaluation of publications. It applies mathematical and statistical tools to measure the inter-relationships and influence of publications within the scientific community [22,23]. This method can provide a macroscopic overview of how the publications have had an impact on the scientific community, which can be determined by the number of times an article has been cited by other authors [24]. The design study of bibliometric analysis maps out the academic output as citation information to assess the impacts of an article [25]. This bibliometric analysis also allows for the representation of information in ways that make relationships more obvious and easier to understand leading to new insight and discovery [26]. Previously, De Souza et al. [27] published a bibliometric study on photosynthesized nanoparticles, and Feng and Chen [28] analyzed the bibliometric on nanocatalyst. The bibliometric studies are used to improve the understanding of a research topic, main issues, developments, identifying leading research groups, networking and leading countries in nanotechnology.

**Figure 2:**
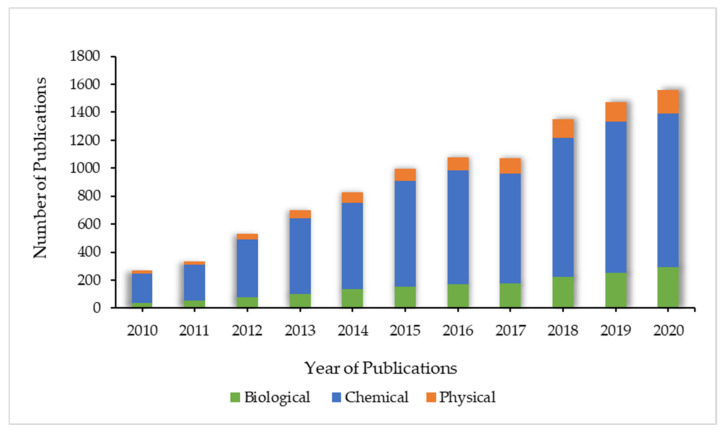
Annual numbers of publications relating to the search terms “Silver nanoparticles” and “Biological/Chemical/Physical” in the Scopus publication database between 2010 and 2020 (English language only).

A bibliometric analysis was carried out to analyze the co-occurrence of keywords. Data base construction was carried out using the Microsoft Excel applied to the Scopus publication database (https://www.scopus.com/ accessed on 26 July 2021). A thematic search was limited to the years 2010 to 2020 pertained within Scopus database using the search term combination (Figure 2): ‘Synthesis’ AND ‘silver nanoparticles’ AND ‘biological synthesis’, ‘Synthesis’ AND ‘silver nanoparticles’ AND ‘chemical Synthesis’, ‘Synthesis’ AND ‘silver nanoparticles’ AND ‘physical synthesis’. The search yielded 951 physical synthesis publications, 7650 chemical synthesis publications and 1677 biological synthesis publications, according to the database. All the data were downloaded on the 26 July 2021. The most popular publication was chemical synthesis, followed by biological synthesis and lastly physical synthesis. Figure 2 also concluded that the number of total publications increases by year except there is a decrease in year 2017.

**Figure 3:**
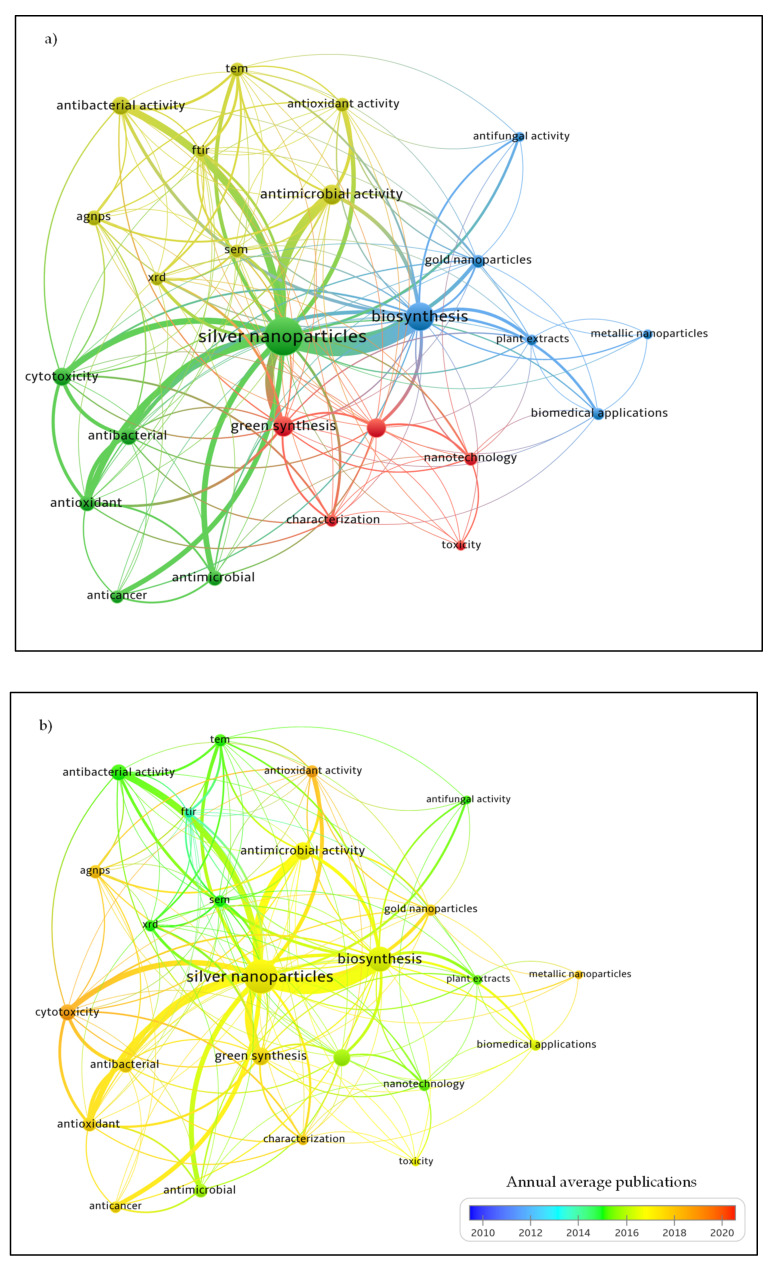
Research trend clusters mapping. (**a**) Network visualization of author keywords co-occurrences (**b**) Network visualization of author’s keywords cluster analysis across the annual average publications on the biomedical applications of AgNPs published between 2010 and 2020. Abbreviations: Fourier-transform infrared spectroscopy (FTIR), Transmission electron microscopy (TEM), Scanning electron microscopy (SEM), and X-ray diffraction (XRD).

The occurrences of author keywords on the biomedical applications of AgNPs were evaluated by using a bibliometric clustering and mapping software, VOSviewer version 1.6.15 (Centre for Science and Technology Studies, Leiden University, Leiden, The Netherlands) [29,30]. The cluster networks were developed using the keywords “biomedical AND application AND biosynthesis AND silver nanoparticles” from Scopus database (Figure 3). A collection of 224 documents were sourced from articles, reviews, book chapters, conference papers and reviews. The similarity could be inferred using this mapping approach based on the strength of co-occurrence or linkage. A stronger co-occurrence frequency is designed to evaluate keyword correlation. The minimal frequency of keyword occurrences was attained with a threshold of five and defined to only 25 keywords. These keywords represented as the terms or words mentioned in the titles, keywords, and abstracts for more than five times in overall 224 documents.

Figure 3a depicts the clusters formed around the central theme of the interested topic. Each keyword is represented by varying sizes of nodes (circle). The number of documents published between two keywords measured by the total strength of link (TLS). Thickness of line connecting one node to another, indicates the keyword’s co-occurrences. The strength of the correlation link is determined by the distance between each keyword. A stronger relationship is revealed by a smaller distance, vice versa [29,31,32]. The overlay visualization map is straightforward, and the impact of each keyword was presented from a decade literature collection (Figure 3b) [33]. The author keywords were displayed by the nodes on a network map with a colour gradient based on the software’s assessments for the average publishing year. Keywords were distributed according to average publication year from earliest to recent with blue (lowest score) to red (highest score) colour ranges. Keyword analysis could be performed to discover the underlying areas in the study of biomedical applications of biosynthesis of AgNPs. This would represent as an enhanced resource for scientific research and efficiently extrapolate the core of subjects in a disciplinary field of study [34,35].

The classification of keywords into topic areas or research themes is represented by clusters. Four clusters were illustrated in Figure 3a. Cluster 1 (yellow) with 8 occurring keywords linked with “antimicrobial activity” (25 occurrences), “antibacterial activity” (19 occurrences), “AgNPs” (13 occurrences) and “antioxidant activity” (11 occurrences). The most common analytical tools for AgNPs characterization were identified in the cluster as “TEM”, “SEM”, “FTIR” and “XRD”. The largest node formed in Cluster 2 (green) was “silver nanoparticles” which also acts as the central theme in this research topic. The node was linked by “antibacterial”, “anticancer”, “antimicrobial”, “antioxidant” and “cytotoxicity” keywords. The terms “antifungal activity”, “biosynthesis”, “gold nanoparticles”, “plant extracts” and “metallic nanoparticles” highly linked to “biomedical applications” (8 occurrences) were demonstrated in Cluster 3 (blue).

**Figure 4:**
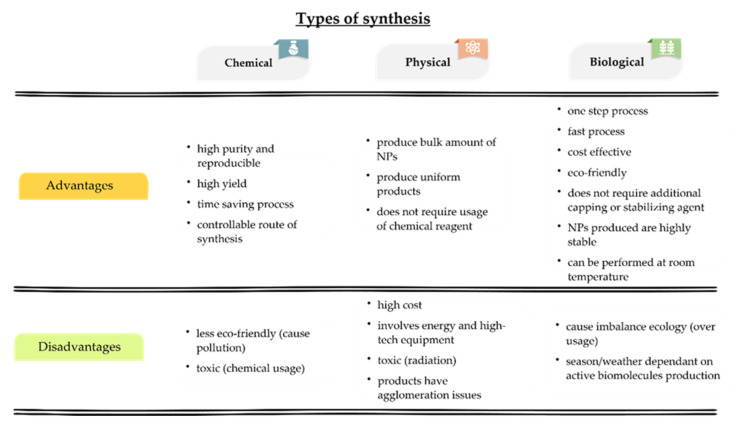
The advantages and disadvantages of three types of synthesis (adapted from Mukherjee and Patra, [38], Simões et al. [39] and Xu et al. [4]).

Moreover, in the Cluster 4 (red) illustrated the keywords “green synthesis”, “nanoparticles”, “characterization”, and “toxicity” commonly occurred in the field of “nanotechnology” (10 occurrences). In Figure 3b, the keyword “FTIR” (7 occurrences) in turquoise colour was term frequently mentioned in articles around the year 2013. The keyword “silver nanoparticles” had the greatest number of occurrences (103) and published by articles between 2016 and 2018. Meanwhile, the keywords, “cytotoxicity”, “AgNPs”, “gold nanoparticles” and “metallic nanoparticles” were regularly contributed in publications towards the year 2020. Considering AgNPs with an outstanding therapeutic potential, much research work has focused into studying the complex processes of their biological functions, possible hazardous consequences, and applications of biomedical technologies for enhanced clinical testing. Figure 4 displays the advantages and disadvantages of all three types of synthesis. The chemical synthesis was preferred due to production of controlled shape and size of AgNPs [36,37].

### 2.1. Chemical Route of Synthesis of AgNPs

The chemical method is the most conventional way to synthesize AgNPs. The medium of synthesis can be organic or aqueous solvent. Chemical synthesis involves the use of toxic and hazardous chemicals that can be potentially harmful to the environment. The chemicals used in synthesis can be flammable and non-biodegradable [40]. These toxic chemicals may be absorbed on the nanoparticles and lead to toxic and adverse effect if used in medical applications [41], example of reducing chemicals used in the chemical synthesis are citrate, ascorbate, element hydrogen and borohydride. Example of chemical synthesis includes chemical reduction, microemulsion technique, sonochemical and microwave assisted synthesis.

#### 2.1.1. Chemical Reduction

The most common method used in chemical synthesis is chemical reduction by organic and inorganic agents. This method is commonly used due to its simplicity. The reducing agents are sodium citrate, sodium borohydride, polyethylene glycol (PVP) and Tollen reagent. These agents are used for reduction of oxidation state from Ag^+^ to Ag^0^ in the aqueous and non-aqueous solutions. The introduction of these reducing agents lead to a rapid rate of reaction, which produced a large amount of metal nuclei that produces extremely small particles [42]. A slow rate of reaction will lead to agglomeration of particles. The reduction in silver nitrate (AgNO_3_) takes place in the presence of stabilizer to shield the growth of NPs through aggregation. It was reported that a specific shape of AgNPs can be synthesized using ascorbic acid, thiosulfate, sodium citrate and polyethylene glycol as reducing agents. The AgNPs produced by these agents are spherical [43]. The usage of a stronger reducing agent produces a smaller AgNPs [44]. Tsuji et al. [45] reported that large AgNPs were produced when weak reducing agent, trisodium citrate was used. The surfactants used were citrate, polyvinyl alcohol, cetyltrimethylammonium bromide, and PVP. Surfactants are used to protect particles from agglomeration and to stabilize the particles. Surfactants can also control morphology of synthesized AgNPs [6,46,47]. Dang et al. [48] reported that a surfactant such as PVP can act as a size controller through electrostatic attraction between the ester bond of surfactant with Ag, thus helping to stabilize and keep the AgNPs smaller in size.

A study by Suriati et al. [42] carried out an investigation on the reduction of AgNO_3_ by ascorbic acid and trisodium citrate as surfactant. They concluded that the increase of trisodium citrate concentration produced more uniform quasi-spherical shape AgNPs. In contrast, when ascorbic acid concentration was increased, a slight change in shape of synthesized AgNPs were observed, from quasi-spherical to polygonal shape. It was also found that increased concentration of trisodium citrate resulted in decrease of AgNPs size, while increased concentration of ascorbic acid showed the opposite result. Ho and Nga [49] have used sodium citrate as reducing agent to synthesize AgNPs. They found that chemical reduction without any surfactant or stabilizer produced AgNPs with good distribution and 20 nm in size. In other studies, AgNPs were synthesized using chemical reduction method with reducing agent, sodium borohydride and stabilizing agent, PVP and sodium chloride [50]. The synthesized AgNPs were 20 nm in size.

#### 2.1.2. Microemulsion Technique

Microemulsion is the synthesis of AgNPs when surfactant is used to thermodynamically stabilize the dispersion of two immiscible liquids such as oil in water or water in oil or water in superficial carbon dioxide, a mixture of oil, surfactant and water or oil, co-surfactant, surfactant, and water. The AgNPs synthesized are uniform in size and shape. Many types of surfactants such as anionic, cationic, and non-ionic surfactants are available to form microemulsion for AgNPs synthesis. Examples of anionic surfactant used are sodium dodecylbenzene sulfonate (SDS), bis(2-ethyhexyl) sulfosuccinate, lauryl sodium sulphate, cationic surfactant are PVP and cetyltrimethylammonium bromide and the non-ionic surfactant is Triton X-100 [43].

AgNPs are synthesized in a two-phase organic aqueous system [51]. Interphase interference and the intensity of interphase transport between phases are mediated by ammonium salt, which affects the rate of interaction between metal precursors and reducing agents. Metal clusters are stabilized in a non-polar aqueous medium and transferred to an organic medium using a stabilizing agent.

Reyes et al. [52] demonstrated the microemulsion synthesis of AgNPs using toluene as organic phase, AgNO_3_ solution and a mixture of surfactants sodium dodecyl sulfate/sodium bis (2-ethylhexyl) sulfosuccinate. Studies were done using different concentrations of precipitating agent, sodium borohydride and dosing times. All medium with higher concentrations of precipitating agents produced worm like nanostructures, whereas the lowest concentration produced a mixture of AgNPs and worm like nanoparticles. In another study, Das et al. [53] reported the synthesis of cubic AgNPs with the size of 17.89 ± 8.74 nm using biosurfactant extracted from *Pseudomonas aeruginosa* MKVIT3 and borohydrate as reducing agent. Similarly, Chen et al. [54] reported that microemulsion method was used to synthesize AgNPs with silver acetate and reducing agent oleylamine at 70 °C. The synthesis produced highly monodisperse AgNPs from 10–20 nm and storage stability of 6 months.

#### 2.1.3. Sonochemical Method

Sonochemical method is the synthesis of AgNPs by ultrasonic radiation, which produced a local hot spot. This method was initially used to synthesize iron NPs, but it has since been applied to the synthesis of a variety of different metals, including Ag [55]. There are several methods for sonochemical synthesis, the most important of which are the formation, growth, and collapse of bubbles [56]. When solutions are exposed to ultrasonic radiation, acoustic fields may cause the bubbles in the solution to implode. The collapse of the cavitation bubble generates a shock wave, which has a rapid impact on the particles’ surface [57]. Temperature, pH, pressure, microjet speed, and cooling rate all influence the synthesis [4]. With this method, the AgNO_3_ solution and nitriloacetate mixture produced spheres, rods, and dendrite shaped AgNPs. The advantages of this method include its simplicity and the ability to control the size of AgNPs by using different precursor concentrations [58].

Kumar et al. [59] used 1 mM AgNO_3_ with different starch concentrations (25 and 30 mg) as reducing and stabilizing agent. Result showed that 25 mg starched AgNPs were smaller than 30 mg, due to aggregation of starched AgNPs at higher concentration. Elsupikhe et al. [60] reported the synthesis of AgNPs in stabilizing agent, κ-carrageenan with different concentration of AgNO_3_ using sonochemical method. The number of AgNPs synthesized increased as the concentration of AgNO_3_ increased. In another study, Patil et al. [61] used sonochemical synthesis to develop AgNPs coated cotton fabrics. The mixture of AgNO_3_, hexamethylenetetramine as reducing agent and PVP as the polymer stabilizer. The hydrodynamic diameters of AgNPs were found increase from 32 to 144 nm as the irradiation time increased 30 to 90 mins. Kuntyi et al. [62] demonstrated sonochemical synthesis using silver sacrificial anode with the sodium polyacrylate solution. The parameters tested were temperature and sodium polyacrylate concentration. The rate of synthesis increases as the temperature and concentration increase.

#### 2.1.4. Microwave Assisted Synthesis

Microwave heating was discovered in the early 1940s and is now used in a variety of applications in the food industry. Microwave assisted synthesis has recently gained attention due to its superiority in synthesizing AgNPs when compared to conventional heating. Microwave-assisted synthesis can also be performed when a mixture is mixed thoroughly and microwaved. Microwaves generate homogeneous heating, resulting in the formation of fine, narrow and uniform sizes of nanocrystals. According to Nadagouda et al. [63], AgNPs synthesized via microwave assisted synthesis produced AgNPs which are smaller, narrow size distribution and have higher degree of crystallization compared to AgNPs synthesized via conventional oil bath heating. This method demonstrated improved control over the nucleation and growth steps in the synthesis of AgNPs [64]. Additionally, microwave-assisted synthesis can provide rapid and uniform heating, reducing the time required for synthesis. Microwave-assisted synthesis can also be used to accelerate the synthesis process following plant-mediated biosynthesis. Renugadevi et al. [65] synthesized AgNPs using biological synthesis, extract of *Baliospernum montanum* followed by microwave heating. Similarly, AgNPs were synthesized using aqueous rhizome extract of *Alpinia galanga* medicinal plant with the aid of microwave irradiation [66]. Abboud et al. [67] synthesized AgNPs using aqueous onion (*Allium cepa*) extract with the aid of microwave heating.

Navaladian et al. [68] synthesized AgNPs by decomposing silver oxalate in a glycol medium using microwave assisted synthesis and PVP was used as the capping agent. The synthesis was carried out using microwave for 60 and 75 s and it produced AgNPs with the size of 5–6 nm and around 30 nm, respectively. Kumar et al. [69] demonstrated the synthesis of AgNPs using glucose and starch as stabilizers. The conversion rate from Ag ions to AgNPs was recorded 99 ± 1% and AgNPs synthesized were spherical and less than 10 nm in size. Another study demonstrated the synthesis of AgNPs using ethanol as a reducing agent and PVP as a stabilizing agent [70]. The AgNO_3_ solution in ethanolic medium was microwaved and produced spherical AgNPs with mean diameter of 10 ± 5 nm. Chung et al. [71] reported the microwave assisted synthesis of AgNPs using starch as a stabilizing agent and ribose and arabinose as reducing agents. Both reducing agent ribose and arabinose produced AgNPs from 15 to 80 nm in size. A complete conversion was achieved by L-arabinose, D-arabinose and D-ribose were after 1 min, 2 to 8 min and 2 to 5 min respectively.

### 2.2. Physical Route of Synthesis of AgNPs

Physical method for synthesis of AgNPs includes evaporation-condensation, laser ablation, solvated metal atom deposition (SMAD) method and ball milling and gamma irradiation [72,73,74,75]. The most important physical methods are evaporation-condensation and laser ablation [76,77].

#### 2.2.1. Evaporation–Condensation

The evaporation–condensation method requires the use of an atmospheric pressure tube furnace or a small ceramic heater to synthesize AgNPs. This technique is frequently used to synthesize AgNPs. The evaporation-condensation process is comprised of three major steps: (a) Material is evaporated or sublimated to form a vapour phase; (b) Material is transported from the source to the substrate; and (c) Particles and/or films are formed through nucleation and then followed by growth. The vapour rapidly cools, forming small AgNPs in high concentrations [76]. Additionally, this method required a specific kilowatt of power from a typical furnace and a certain amount of time to reach a stable temperature. Additionally, the synthesis of AgNPs requires the use of radiation as a reducing agent rather than a hazardous chemical [72]. This method was used to synthesize nanospheres from a variety of metal materials, including Ag [78]. The disadvantages of evaporation-condensation include the lengthy duration of the process and the enormous amount of energy required [15].

In a study conducted by Kruis et al. [78], evaporation- condensation method involves heating of mixture of AgNO_3_ and sodium acetate in a tube furnace. This resulted in conversion of the liquid mixture into gas state, and lastly condensed into AgNPs after the cooling process. The size of AgNPs produced was 3 to 50 nm. Raffi et al. [79] demonstrated the synthesis of AgNPs with an evaporation-condensation method using inert gas, helium in the process chamber. The authors concluded that smaller AgNPs with less agglomerations produced at a lower evaporation temperature and inert gas pressure. The AgNPs produced were spherical and 9–32 nm in size. In another study, Jung et al. [80] reported the synthesis of AgNPs using ceramic heater, which can reach up to 1500 °C to synthesize AgNPs through evaporation-condensation method. The result showed that polydisperse AgNPs were synthesized from a constant temperature of the heater surface. The size of AgNPs produced was 6.2 to 21.5 nm. In addition, the AgNPs were spherical and non-agglomerated. Similarly, Harra et al. [81] have synthesized AgNPs using furnace at temperatures between 1300 and 1400 °C and the vapor was diluted with nitrogen, N_2_ gas. The size of AgNPs produced was 50, 90 and 130 nm at different temperature of synthesis.

#### 2.2.2. Laser Ablation

The laser ablation method is a method to obtain metal colloids without using chemical reagents. In this method, intense laser pulses are focused on Ag target immersed in a solvent [82]. AgNPs could be synthesized via laser ablation of metallic bulk materials in solution [83]. Other than that, the length of chain in alcohol also plays a role in size of AgNPs synthesized. The alcohol with a longer chain length from C-3 to C-5 synthesized more stable and smaller particles than short chain alcohols such as methanol and ethanol [84]. The characteristics of the AgNPs formed are influenced by a few factors including the wavelength of laser, the duration of laser pulses (femto-, pico- or nanoseconds), the laser fluence, ablation time effective medium and the presence of surfactants [51]. The longer the timing of ablation, the higher the concentration of AgNPs until saturation value is reached [85]. Increasing surfactant concentration can also produce smaller AgNPs [51]. A study showed that femtosecond laser pulses produced AgNPs with narrower size distribution compared to nanosecond laser pulses [45]. After the ablation, the liquid environment only contains AgNPs without other ions, reducing agents and compounds [86].

Amendola et al. [87] synthesized AgNPs in acetonitrile and *N*, *N*-dimethylformamide using a bulk metal with laser ablation. The synthesis produced AgNPs surrounded by a carbon shell or included in a carbon matrix. Tajdidzadeh et al. [88] have synthesized AgNPs in ethylene glycol and chitosan solution. The method used chitosan solution produced smaller AgNPs with a longer stability compared to AgNPs in ethylene glycol. In a similar study, AgNPs were synthesized with isopropanol coating using laser beam have had several month stabilities [89]. Alhamid et al. [90] have produced AgNPs in distilled water by laser ablation with continuous shots for 13 h. Menazea [91] investigated the synthesis AgNPs with different liquid media such as deionized water, distilled water, dimethylformamide and tetrahydrofuran. The result reported that AgNPs synthesized in deionized water have higher ablation, stability and antibacterial efficiency than other media. Rhim et al. [92] studied the preparation of AgNPs by a laser ablation method in 5% PVP and a square silver plate was used in process.

#### 2.2.3. Solvated Metal Atom Deposition (SMAD) Method

SMAD occurs when a bulk metal is evaporated under a vacuum to produce atoms or small clusters of Ag, while controlling the aggregation of these clusters [93]. The vapour of the metal then co-condensed with vapours of organic solvents such as acetone to form NPs in solution using a physical method. A metal wire under vacuum is then electrically heated to achieve the evaporation of metal. The resulting solution would contain only colloids and solvent with no byproducts.

The size of produced nanocrystals can be tuned by varying variables such as solvent polarity and heating rate [94]. The advantages of this method include the absence of a reduction step, the ability to use a wide variety of metal-solvent combinations, the avoidance of toxic organometallic compounds, the achievement of very high dispersion of zero valent metals, the frequent encounter of unusual metal morphology that is highly reactive, and the secure attachment of metal particles to the catalyst support.

The AgNPs synthesized were in various sizes and spherical in shape. Similarly, Baudot et al. [95] reported the AgNPs and other metal NPs were synthesized using SMAD method with chitosan was used as a capping agent. The AgNPs synthesized with chitosan resulting in an average size of 6 ± 1.3 nm.

#### 2.2.4. Ball Milling

Mechanical milling is another term for ball milling. John Benjamin and his colleagues at the International Nickel Company invented high-energy ball-milling in the late 1960s [96]. Ball milling is a type of physical synthesis that involves dropping a ball into a container and rotating it horizontally, which produces smaller AgNPs. Smaller AgNPs have a higher surface energy, which results in particle aggregation [4]. There are several types of mechanical mills that are used in synthesis: vibratory, attritor, planetary, and uniball mills [55]. The dispersion quality is determined by the milling time, rotational speed, and ball size [74]. Diffusivity and phase of synthesized AgNPs are temperature dependent during the ball milling process [97]. The quality of ball-milled products is influenced by a variety of factors, including the milling speed, time, temperature, atmosphere, process control agent, ball to powder ratio, and the size and size distribution of the grinding medium [98].

Cryomilling involves high energy ball milling and able to produce AgNPs in large quantities at low temperature (−160 ± 10 °C). AgNPs produced were 4–8 nm in size and the Ag powder collected was cooled down by LN_2_ [99]. Rak et al. [100] synthesized AgNPs using lignin as reducing agent and polyacrylamide polymer as support polymer. The mixture of lignin, polyacrylamide and AgNPs was milled for 1.5 h and the AgNPs produced were 1 to 30 nm. Khayati and Janghorban [97] demonstrated the synthesis of AgNPs graphite as reducing agent using mill. AgNPs synthesized were 14 nm in size with the presence of process control agents.

#### 2.2.5. Gamma Irradiation

Gamma irradiation has high energy, which can be used to synthesize AgNPs [101]. Gamma irradiation is a method by which the Ag^+^ solution induces the reduction of Ag^+^ into metallic Ag. The colour of irradiated samples changes from colourless to golden yellow for irradiated Ag [102]. In addition, the reducing agent used can be uniformly distributed in the solution and the AgNPs synthesized are highly pure and stable [103].

Rao et al. [104] reported the production of AgNPs using gamma radiation and gum acacia as a protecting agent. Smaller AgNPs produced due to higher concentration of gum because it contributes to enhance steric stabilization. Leawhiran et al. [105] had prepared antibacterial hydrogel wound dressing contained AgNPs using polyvinyl alcohol (PVA), gelatine and AgNO_3_ solution through gamma irradiation. Eghbalifam et al. [106] investigated the synthesis of AgNPs in the mixture of PVA and sodium alginate under different gamma ray doses. A higher amount of AgNPs with smaller size was formed as the irradiation dose increased. However, it was observed that no PVA hydrogel was formed up to 15 kGy radiation. In another study, Madhukumar et al. [107] described the synthesis of AgNPs in aqueous silk fibroin solution obtained from *Bombyx mori* silk using different gamma irradiation dose. The results showed that 30 kGy, 40 kGy and 50 kGy irradiated samples produced 20, 33 and 22 nm in size of AgNPs respectively. Pratiwi et al. [101] synthesized AgNPs with PVA mixture and 10 mM AgNO_3_ solution under different gamma irradiations, which were 0, 0.5, 1 and 2 kGy. The authors concluded that the higher radiation dose increase absorption intensity.

### 2.3. Biological Route of Synthesis of AgNPs

Biological synthesis of NPs is the simplest, non-toxic, and yet most ecofriendly method for producing high-quality NPs. In comparison to chemical and physical synthesis, biological synthesis of AgNPs is advantageous. To synthesize NPs via biological synthesis, safe, non-toxic, and environmentally friendly reagents are used. Additionally, AgNPs synthesized in a single step, such as biological synthesis, have a higher degree of stability, diversity, and adequate dimensions [7]. Biological route synthesis requires living organisms such as bacteria, fungi, plants and algae. In addition, extracts from organisms can act as reducing and capping agents in AgNPs synthesis [108]. The reduction of Ag ions is facilitated by biomolecules such as enzymes, proteins, polysaccharide, amino acid and vitamins that can be found in the extracts and they are biodegradable.

#### 2.3.1. Bacterial-Based Biosystems

Bacterial-based biosystems have been reported to synthesize AgNPs either intracellularly or extracellularly. AgNPs produced by microorganisms can be classified into two distinct categories, depending on the location where they were produced. AgNPs generally can be produced by bacteria by bioreduction process, in which reduction of AgNO_3_ precursor from Ag^+^ ions to Ag^0^ forming AgNPs by Nicotinamide adenine dinucleotide (NADH)-dependent enzymes [109]. Singh et al. [110] synthesized AgNPs using *Pseudomonas* sp. THG-LS1.4. Thomas et al. [111] synthesized AgNPs intracellularly using *Ochrobactrum anhtropi*. However, extracellular biosynthesis mode was preferred due to easy recovery of AgNPs.

The synthesized AgNPs exhibit a range of shapes and sizes (Table 1). The type of bacteria used, the temperature, pH, and substrate concentration all have an effect on the shape and size. Numerous researchers have demonstrated that bacteria are capable of synthesizing silver and other NPs. The synthesis of AgNPs is a bioreduction process [109]. Studies revealed that bacteria produce extracellular reductase enzymes to reduce silver ions to AgNPs. Researchers revealed NADH-dependent enzymes involved in bioreduction and the reductase enzymes receive their electrons from NADH. The NADH is then oxidized into NAD^+^ ions.

Wang et al. [133] investigated the synthesis of AgNPs using *Bacillus methylotrophicus*. They reported that the AgNPs synthesis was carried out after 48 h of incubation at 28 °C. The synthesized NPs were tested against *Candida albicans*, *Escherichia coli*, *Salmonella enterica* and *Vibrio parahaemolyticus*. The results showed a better inhibition growth compared to antibiotics. Similar study was done by Karunakaran et al. [134], by performing synthesis of AgNPs using *Aztobacter vinelandii* culture extracts and spherical NPs with an average size of 20–70 nm were produced. These AgNPs exhibited a strong antioxidant activity and antibacterial properties against pathogens such as *S. aureus*, *E. coli*, *S. fradiae* and *S. marcescens*. The author also suggested that these AgNPs can be efficiently used in the development of nanomedicine. These AgNPs are efficiently used in health, food, and pharmaceutical industry.

Otari et al. [135] synthesized AgNPs using *Rhodococcus* sp. after 10 h incubation at room temperature. The AgNPs synthesized were spherical shape with 10–12 nm in size and they showed both bactericidal and bacteriostatic activity against wide range of bacteria. In another study, endophytic bacterium *Bacillus siamensis* C1 was isolated from a medicinal plant *Coriansrum sativum* and AgNPs synthesized were about 25 to 50 nm in size [136]. These NPs showed a strong inhibitory property against rice bacterial leaf blight and bacterial brown stripe as well as promotes plant growth. *Penicillium brevicompactum* (MTCC-1999) was used to synthesize AgNPs, which were found to be spherical in shape and ranging in size from 30 to 50 nm [137]. The AgNPs exhibited excellent antibacterial and anticancer activity, with IC50 values of 70 and 50 μg/mL after 24 and 48 h, respectively. Wpij et al. [138] investigated the synthesis of AgNPs using an actinobacterial reductant. The biosynthesized AgNPs demonstrated antibacterial activity against *E. coli*, *K. pneumoniae*, *P. aeruginosa*, and *S. aureus*, as well as anticancer activity against RAW 264.

#### 2.3.2. Use of Fungi

Fungi are well-known for their ability to decompose organic matter. Recent data indicate that approximately 5.1 million fungal species exist [139]. Around 6400 bioactive substances are produced by filamentous fungi and other fungal species on a microscopic scale [140]. Fungi can synthesize AgNPs intra- or extracellularly, depending on the location of the synthesized AgNPs. Certain fungi are capable of synthesizing AgNPs in both ways [141]. Fungi reduce Ag^+^ ions to Ag^0^ forming AgNPs. NADH and NADH-dependent nitrate reductase are involved [142,143]. Fungi secretes extracellular enzymes such as protease, cellulase, chitinase and β-glucosidase to degrade cellulose, protein, starch, hemicellulose, and animal compounds as a food source [144]. Fungi such as lignocellulolytic fungi obtained their source of carbon and energy by secreting extracellular hydrolase and oxidase enzymes to break down lignocellulosic materials, such as cellulose, lignin and hemicellulose [145]. Fungi are fancied as they secrete a large number of proteins and enzymes, which directly fasten and increase the synthesis of AgNPs [146,147]. Due to the ability to tolerate and metal bioaccumulate, fungi have attracted more attention in the biological production of AgNPs [143]. The biosynthesis involving fungi is usually free from toxic chemicals. Most fungi have high wall-binding and intracellular metal uptake, whereas several species grow fast, thus making it easy to culture and keep them in a laboratory [148]. Filamentous fungi have gained more attention compared to other fungi because the AgNPs synthesized by them have good morphological characteristics, stability, and a wide range of applications. Fungi have the ability to synthesize nanoparticles both intra- and extracellularly. Intracellular synthesis results in nanoparticles that are smaller than those synthesized extracellularly [149]. Intracellular is preferred for a variety of reasons, which includes impurities contamination from enzymes samples can be easily extracted, as the intracellular enzymes generally require fewer purification steps [150]. Some metal ions could induce specific proteins, which can hydrolyze the ions.

In a study, *Aspergillus sydowii* was used to synthesize AgNPs, with spherical shape and size ranging from 1 to 24 nm [151]. These NPs showed antiproliferative and antifungal properties against clinical pathogenic fungi, which can be applied in biomedical applications. *Beauveria bassiana* fungus biosynthesized AgNPs with a mixed size and shape, ranging from 10 to 50 nm with the mixture shapes of circular, triangular, and hexagonal shape [152]. It was concluded that polyphenolic components were released by fungus used for NPs synthesis and growth. Polyphenols also act as a capping agent in the synthesis [153].

Husseiny et al. [154] synthesized AgNPs using fungi, *Fusarium oxysporum* and evaluated the effects on cancer cells. In addition, these AgNPs were also effective as antimicrobial potential against *E. coli* and *S. aureus*. El-Alziz et al. [155] studied the synthesis of AgNPs using fungus *Fusarium solani* isolated from wheat and these NPs have antifungal effect against different species of phytopathogenic fungi that contaminates wheat, barley, and maize seeds. Sundaravadivelan and Padmanabhan [156] investigated use of synthesized AgNPs from *Trichoderma harzianum* to control insect vectors. Authors have concluded that the synthesized AgNPs were effective against controlling the pupae and larvae of *Aedes aegypti*. Authors also suggested that these NPs could be used as an inexpensive approach to control *A. aegypti*. Elgorban et al. [157] demonstrated a study using fungus *Aspergillus versicolor* to synthesize AgNPs. An anti-pest study was conducted against *Sclerotinia sclerotiorum* and *Botrytis cinerea* in strawberry plants using the AgNPs synthesized. The result concluded that AgNPs showed greatest effect against *B. cinerea*.

A green synthesis utilizing the filamentous fungus *Aspergillus fumigatus* produced AgNPs with a diameter of 5–25 nm [158]. Gajbhiye et al. [159] synthesized polydisperse spherical AgNPs ranging in size from 20 to 60 nm and evaluated their antifungal activity against *Phoma glomerata*, *Phoma herbarum*, *Fusarium semitectum*, *Trichoderma* sp., and *Candida albicans*. The combination of fluconazole and synthesized AgNPs inhibited *C. albicans* the most, followed by *P. glomerata* and *Trichoderma* sp., with a smaller increase in fold area of inhibition, but no enhancement against *P. herbarum* or *F. semitectum*. Vigneswaran et al. [160] reported that *Aspergillus flavus* synthesized monodispersed AgNPs with a size of 8.92 ± 1.61 nm and used them to study the bioabsorption of Ag as NPs. The AgNPs synthesized were found to be more than three months stable in water. Another study demonstrated that the endophytic fungus *Botryosphaeria rho-dina* synthesizes spherical AgNPs ranging in size from 2 to 50 nm. It was reported that synthesized AgNPs exhibited in vitro anticancer efficacy against A-549 cells with an LC50 of 40 g/mL [161].

Numerous reports have indicated that NADPH-dependent reductase and electron transfers may be involved in the synthesis of AgNPs. Hulikere and Joshi [162] reported that even small molecules such as phenolics may be involved in the synthesis of AgNPs when marine endophytic fungi, *Cladosporium cladosporioides* species, are used. This was confirmed by the authors’ FT-IR analyses. *S. aureus*, *S. epidermis*, *B. subtilis*, *E. coli*, and *Candida albicans* were all inhibited by the AgNPs synthesized. Kosbashigawa et al. [163] used *Trametes trogii* to synthesize AgNPs and obtained a wide variety of AgNPs sizes. Additionally, the authors hypothesized that several ligninolytic enzymes are involved in this biosynthesis process.

#### 2.3.3. Use of Plants

**Figure 5:**
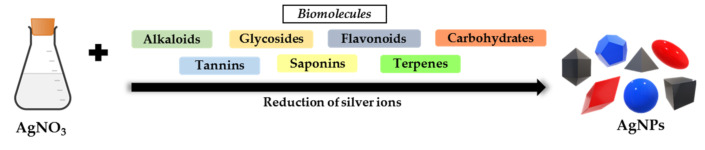
Biomolecules in flower extracts of *Ixora coccinea* involved in the synthesis of AgNPs.

Biological synthesis methods that utilize plants as an AgNPs synthesizer are superior to other biological methods because plants are abundant in comparison to other biological resources [164]. Additionally, when compared to biological synthesis using microorganisms, using plants to synthesize AgNPs reduces isolation costs, culture media costs, and environmental contamination [165,166]. It has been demonstrated to have a slower kinetic and a greater ability to manipulate crystal growth and stabilization. Green plants provide a rapid, eco-friendly, cost-effective, non-pathogenic, and one-step method for AgNPs synthesis [167]. AgNPs are synthesized from plant extracts including leaves, stems, roots, seeds, and latex [168,169,170]. The shape, size, and concentration of AgNPs produced by plants are determined by their chemical composition and concentration, AgNO_3_ concentration, extraction solvent used, extraction time and temperature, and reaction time and temperature [171]. Nalvolthula et al. [172] summarized the synthesis of AgNPs, which involves a few types of biomolecules present in the flower extracts of *Ixora coccinea* (Figure 5).

Plant extracts contain biomolecules such as polysaccharides, tannins, alkaloids, amino acids, vitamins, polyphenols, terpenoids and saponins, which are eco-friendly and have medicinal values, that involve in reduction and stabilization of Ag ions [173]. Perera et al. [174] stated that biomolecules such as tannins, flavonoids and many sugars act as reducing agents whereas other biomolecules act as capping or stabilizing agent during AgNPs synthesis. The high demand of AgNPs can be resolved by the large-scale production of AgNPs, which takes place in the plants. The plants leaf is preferred compared to the whole plant in the synthesis of extracellular AgNPs [175].

**Figure 6:**
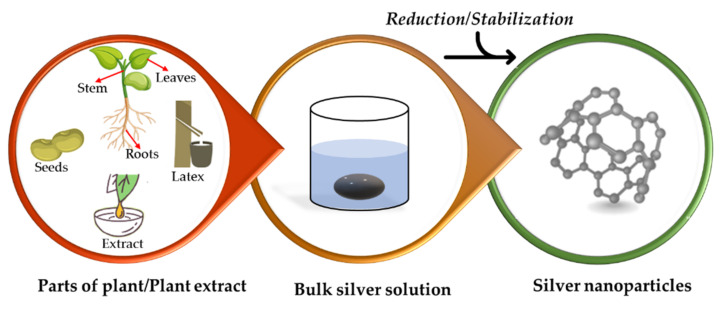
Overview of steps of synthesize mediated by plant or plant extract (adapted from Nadeem et al. [176]).

The proposed hypothetical mechanism for the synthesis of AgNPs (Figure 6) involves plants or extracts of plants containing enzymes and bioreductant molecules that act as reductants when combined with metal to form AgNPs. For instance, a plant extract containing a reducing enzyme will be capable of reducing AgNO_3_ to silver and nitrate ions. Additionally, the complex network of enzymes and antioxidant metabolites acts synergistically to protect the cell and its components from oxidative damage [164].

The volume of plant extract, the silver concentration, the pH, the reaction time, and the pH value of the mixture all significantly influence the size and shape of AgNPs produced [177]. The pH of a mixture has a significant effect on the NP composition. Changes in pH value result in a charge shift in plant metabolism, which has an effect on the chelating and reducing capacity. This can result in variations of the morphology, dimension, and yield of the synthesis. By increasing the pH of the reaction mixture, small and uniform sized particles are produced [178,179,180]. The shape of the AgNPs produced can also be altered from nearly spherical to spherical by adjusting the pH [181].

Concentration of AgNO_3_ also plays an important in AgNPs synthesis. Smaller size of AgNPs produced as the concentration of AgNO_3_ were increased [182,183,184]. Temperature is also one of the essential factors affecting the properties of AgNP during synthesis. High temperature improves the rate of nucleation, which certainly produces smaller AgNPs. Different metabolites have different nature and reduction capability. Polyphenol family is a group that possess all good characteristics compared to other available metabolites. The characteristics are sufficient molar concentration, high reducing/antioxidant power and direct involvement in heavy metal detoxification mechanism in plants. The most abundant polyphenols are flavonoids sub-class. Flavonoids usually have different biological functions such as protection against ultraviolet (UV) radiation and phytopathogens, strong antioxidant activity and flower and fruit coloration [185]. Types of metal ions used also affects the NPs production. The metal ions with higher electrochemical potential are likely to condense faster [186]. For example, Ag ions have a better ionizing potential due to their smaller size compared to Au ions; therefore, it condenses faster.

Jha et al. [187] synthesized AgNPs (2–5 nm) using plant extracts of *Bryophyllum* sp., *Cyperus* sp. and *Hydrilla* sp. It was confirmed that the reduction was due to phytochemicals such as flavones, quinones and organic acid present in plant tissues. In other study, *Solanum indicum Linn* was reported to synthesize AgNPs, which showed larvicidal activity against *Cx. pipiens* as well as antibacterial activity against *S. aureus*, *E. coli*, *P. mirabilis* and *Shigella flexneri* [188]. *Solanum nigrum* and *Clitoria ternatea* were reported to synthesize small AgNPs and exhibited antibacterial properties against *S. aureus*, *B. subtilis* and *S. pyogene* [189]. NPs synthesized by *Clitoria ternatea* extract were smaller in size and exhibited higher antimicrobial activity against nosocomial pathogens compared to NPs synthesized by *Solanum nigrum*, *Citrus sinensis*, *Centella asiatica*, *Syzygium cumini* and *Solanum tricobatum*, which were irregular shape AgNPs of average size 41, 42, 53 and 52 nm. These AgNPs were found effective against *P. aeruginosa* [190]. *Amaranthus gangeticus Linn* leaf extract was able to synthesize globular shape AgNPs, which exhibited antifungal properties as well as antibacterial properties against both gram-positive and gram-negative bacteria [191]. Velmurugan et al. [192] demonstrated the synthesis of AgNPs by *Prunus yedoensis* and found that the synthesized NPs were more effective against skin bacteria than commercial AgNPs.

Saratale et al. [193] reported on the synthesis of AgNPs using *Punica granatum* leaf extract (PGE), which resulted in spherical NPs measuring 35 to 60 nm in diameter. The biosynthesized AgNPs exhibited antidiabetic activity by inhibiting a-amylase and a-glucosidase. Additionally, AgNPs demonstrated significant anticancer activity when tested against human liver cancer cells (IC50; 70 lg/mL). In another study, AgNPs were prepared using an extract of the *Ipomoea pes-caprae* plant, and the AgNPs demonstrated significant antibacterial activity against *P. aeruginosa*, *E. coli*, and *Bacillus*, as well as anticancer activity against MCF-7 cancer cells. The MTT assay confirms the anticancer activity, with an IC50 of 78 g of AgNPs/mL [194].

The plant, *Brassica nigra* was used to synthesize 10 to 50 nm of spherical shape AgNPs. The AgNPs synthesized showed a strong antifungal and antimicrobial properties as well as anticancer activity at 100 μg/mL (IC50 of 55 μg/mL) on cancerous cells and was dose dependent [195]. Kanmani and Scleeva [196] reported the synthesis of AgNPs using *Linumisitatissimum extract* produced needle shaped AgNPs. The AgNPs produced showed antidiabetic activity with a maximum inhibition of 79.84% in alpha-amylase assay and 58.86% for alpha-glucosidase at 100 μg/mL.

#### 2.3.4. Use of Algae

Algae have fast growth rate, cost effective scale-up and easy to harvest. Algae are the earliest and most abundant photosynthetic organisms on the earth [197]. AgNPs can be synthesized in a variety of shapes, including rods, wires, hexagons, spheres, cubes, and pentagons [4]. Additionally, algae are abundant in resources such as pigment, peptides, proteins, and secondary metabolites, which act as nano-biofactories [198]. Green algae (*Chlorophyceae*), blue-green algae (*Cyanophyceae*), red algae (*Rhodophyceae*), and brown algae (*Phaeophyceae*) are all types of algae [12,199,200]. Algae synthesize AgNPs in two distinct ways: intracellularly and extracellularly [120]. Intracellular mode occurs within the cell and does not require any pretreatment. Respiration, photosynthesis, and nitrogen fixation are likely to be involved in the synthesis. NADPH or a NADPH-dependent reductase may be used as the reducing agent. Proteins containing amino groups or cysteine residues serve as stabilizing and capping agents, as do sulfated polysaccharides [201].

**Figure 7:**
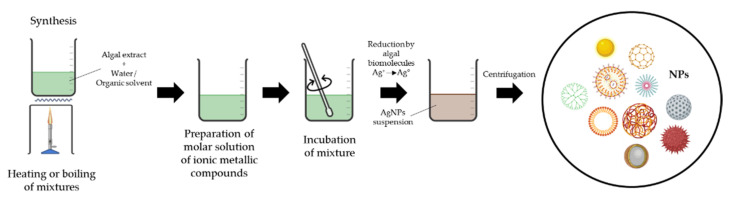
Overview of steps of synthesize mediated by algae extract (adapted from Vincy et al. [202]).

Three steps are required for AgNPs synthesis using algae, as shown in Figure 7: (i.) heating or boiling water or organic solvent and algal extract for a specified duration, (ii.) preparing a metal precursor solution, and (iii.) incubating the precursor with algal extract under controlled conditions with continuous stirring. The colour change of the AgNO_3_-algal extract mixture was then monitored to determine whether AgNPs were formed [119,202,203]. Extracellular mode occurs external to the cell and entails pretreatment steps such as blending and washing. Metabolites, pigments, ions, various proteins (enzymes), and non-protein structures such as RNA, DNA, antioxidants, hormones, and lipids all contribute to the process.

*Spirulina platensis* is a blue-green alga, the major contributor of AgNPs. *S. platensis* is free floating, filamentous cyanobacteria that synthesized spherical AgNPs (2–8 nm) [204]. The algae also contain 60–70% vegetable protein, which is rich in essential beta carotene, iron, amino acid, natural vitamins, and essential fatty acid, which involves in capping and reduction of NPs. These kind of AgNPs are efficiently used in health, food, and pharmaceutical industry.

Husain et al. [205] screened thirty cyanobacterial species for extracellular synthesis of AgNPs. The results showed all 30 strains were able to synthesize AgNPs, with variety of sizes. Majority NPs synthesized were spherical. *Cylindrospernum stagnale* was the best strain producing smallest AgNPs with the size of 38 to 40 nm. *Microcheate* was the fastest strain in synthesizing AgNPs, with the timing of 30 h. Similarly, Singh et al. [206] had reported cyanobacterium *Leptolyngbya* was able to synthesize spherical AgNPs with average size of 20–35 nm. These NPs showed antibacterial properties against both gram-positive (*S. subtilis*) and gram-negative (*E. coli*) bacteria. In addition, these NPs also showed enhancement in seed germination and early seedling development of wheat. Therefore, these NPs might have potential in pharmaceutical and agricultural industries. Other studies have demonstrated the isolation several cyanobacteria from Muthupet mangroves, including *Spirulina*, *Oscillatoria*, *Microcoleus*, *Alphanocapsa, Phormidium*, *Gloecaps* and *Synechococcus* [207]. The AgNPs synthesized were spherical in shape and size about 40 to 80 nm. *Microcoleus* sp. was reported to have enhanced antibacterial activity against pathogens such as *Salmonella typhi*, *Vibrio cholera*, *Proteus vulgaris*, *Streptococcus* sp., *Bacillus subtilis*, *Escherichia coli* and *Staphylococcus aureus*.

More than 20 different species of green micro algae species are exploited for AgNPs synthesis and almost all micro algae species can synthesize various size and morphology of AgNPs [208]. Rajkumar et al. [209] had conducted biosynthesis of AgNPs using microalgae *Chlorella vulgaris* and the AgNPs obtained were about 55 nm in size. *C. vulgaris* also showed the potential of photocatalytic dye degradation and this can be applied in environmental bioremediation to remove harmful dyes from industries. Another study used microgreen algae, *Scenedesmus* sp., to synthesize NPs both intracellularly and extracellularly, yielding AgNPs with an average size of 15–20 nm and 5–10 nm, respectively. Additionally, *Scenedesmus* sp. exhibited a high level of antimicrobial activity against gram-negative and gram-positive bacteria [210]. *Ulva fasciata* is a significant green macroalga which is able to synthesize AgNPs and demonstrating antimicrobial efficacy [211]. Besides that, other species of green macro alga that able to generate AgNPs were *Chaetomorpha linum*, *Gracilaria corticate*, *Gracilaria edulis* [201,212,213]. In another study, AgNPs biosynthesized by red algae, *Gelidium amansii* reported could minimize the micro-fouling. AgNPs synthesized by brown algae like *Padina pavania* were 49 to 86 nm in size and various shape like rectangle, polyhedral, hexagonal, triangular, and spherical [214]. Another brown algae, *Padina tetrastromatica*, was reported to synthesis spherical AgNPs, which exhibited antimicrobial activity against *B. subtilis*, *K. planticola*, *Bacillus* sp. and *Pseudomonas* sp.

Venkatesan et al. [215] described a green synthesis of AgNPs using the marine algae *Ecklonia cava*, which resulted in spherical AgNPs with an average size of around 43 nm. Antioxidant activity, antibacterial activity against *E. coli* and *S. aureus*, and anticancer activity against human cervical cancer cells were demonstrated for the synthesized AgNPs. *Sargassum muticum*, another brown algae, was used to synthesize AgNPs, yielding 40–65 nm NPs with spherical and hexagonal shapes. At doses of 25 and 50 g/mL of AgNPs, the produced AgNPs demonstrated effective anticancer properties against the breast cancer cell line (MCF7) [216]. Kiran and Murugesan [217] demonstrated the synthesis of AgNPs using the marine alga *Colpomenia sinuosa*, with the particles ranging in size from 54 to 65 nm and having a cubic shape. Antidiabetic activity of biosynthesized AgNPs against alpha-glucosidase and alpha-amylase showed 90.50 ± 0.10 and 94.30 ± 0.10, respectively at concentration of 1 mg/mL.

### 2.4. Characterization of AgNPs

**Figure 8:**
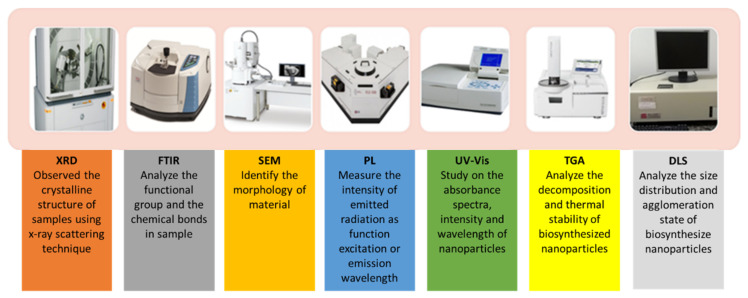
Methods of characterization of biosynthesized AgNPs.

The unique properties of AgNPs will determine their potential and application. A variety of measurement techniques can be used to characterize AgNPs nanoparticles. Researchers employ a variety of classification schemes. Occasionally, researchers classified the characterization of nanoparticles they prepared according to their structural, optical, or electrical properties. Nanoparticle structural characterization is further subdivided into morphology, crystal structure, and composition. Additionally, there are classifications for spectroscopy and microscopy techniques. UV-Visible (UV-Vis), Fourier Transformation Infrared (FTIR), Dynamic Light Scattering (DLS), Energy Dispersive X-Ray Analysis (EDX), and Photoluminescence (PL) are examples of spectroscopy techniques that are frequently used to characterize nanoparticles. The scanning electron microscope (SEM), the transmission electron microscope (TEM), the atomic force microscope (AFM), and the high-resolution transmission electron microscope (HRTEM) are all examples of microscopic techniques that are frequently used by material science researchers. Several of the characterizations listed above are required for biosynthesis-based nanoparticles. Among the listed techniques, FTIR, DLS, XRD and TEM are thoroughly explained in the following subsection. The required comprehensive characterization techniques for the biosynthesis of AgNPs are depicted in Figure 8.

#### 2.4.1. Fourier Transform Infrared Spectroscopy (FTIR) Analysis

FTIR is a method utilizing infrared light to characterize the structure of matter at the molecular scale. This method displays various chemical bonding in a sample or materials as well as detects contaminants in a material, identifies oxidation and decomposition and finds additives [95]. A typical FTIR includes IR source, mirrors, beam splitter, detector and a computer. The IR radiation emitted from the source hits the beam splitter is partly directed to two different mirrors. The two mirrors are the moving mirror, which moves at a constant velocity during data acquisition, and a stationary mirror. The IR beam reflects and recombines at the beam splitter then passed through the sample. The infrared that passes through the sample is usually 10,000 to 100 cm^−1^. The radiation is the absorbed by the sample and converted to rotational or vibrational energy before reaching the detector. The data obtained are processed by a computer to transform the interferogram produced into an IR spectrum.

Recent studies on characterization of AgNPs synthesized by Cannonball leaves and the FTIR showed three prominent peaks at 2927, 1631 and 1383 cm^−1^ [217]. The sharp and strong peak at 1631 cm^−1^ corresponded to stretching vibration of (NH) C = O group, peaks at 2927 was methoxy compounds and 1383 cm^−1^ was C-C and C-N stretching. Another study was on the characterization of AgNPs using FTIR reported that many absorption bands observed [218]. There were bands at 3422 cm^−1^, which related to the presence of alcohol and phenol. There were bands at 2921 and 2856 cm^−1^ represented aromatic compounds, and 1450 and 1043 cm^−1^ represented amine stretch vibration of proteins. Studies of characterizing AgNPs synthesized by *Trichoderma longibrachiatum* using FTIR showed many bands, which were at 1634.92, 2156.94 and 3269.31 cm^−1^. The band 1632.92 cm^−1^ represents the stretching vibrations of primary amines [219].

#### 2.4.2. Dynamic Light Scattering (DLS)

DLS is also known as Quasi-Elastic Light Scattering (QELS) or Photon Correlation Spectroscopy [220]. DLS is a technique for determining the size and distribution of hydrodynamic particles in a range of sizes. Currently, DLS is one of the quickest and most popular methods for determining the size of particles in the range of 1 nm to 1 μm [70,221,222]. Additionally, it is cost-effective and timesaving, as it is a rapid analyzer. The DLS technique is used to determine changes in the intensity of scattered light from a suspension or solution caused by Brownian motion. Brownian motion is the random movement of particles in a zigzag pattern from any direction. According to Brownian motion analysis, larger particles move slower, encompass a shorter distance, and scatter more light than smaller particles. The size and shape of the macromolecules influence the hydrodynamic diameters [223]. The larger particles scatter more light than the smaller ones, whereby even small amounts of aggregates or dust particles could shift the particle size distribution to a larger value. The velocity of the Brownian motion is defined by the translation diffusion coefficient (*D*) and can be converted into a particle size using Stokes-Einstein equation [224]. The equation is as below:

***D*** = ***k_B_*****T**    **3ηπ*d*** (1)

where *D* = diffusion coefficient (m^2^s^−^^1^), *d* = hydrodynamic diameter, *k_B_* = Boltzmann’s constant (1.38 × 10^−^^23^ NmK^−^^1^), T = temperature (K), η = solvent viscosity (N s m^−^^2^).

The polydispersity index (PDI) is used in size distribution range of the particles with values lie between 0 and 1. The value 0 indicates the presence of highly homogeneous NPs, while and 1 indicates the presence of highly heterogeneous NPs population [225].

A typical DLS includes a laser, detector, digital signal processor correlator and computer. The laser is emitted from the laser light source and illuminates the sample in the cell in the DLS instrument. The scattered light signal is then collected using one of two detectors, one at a 90° scattering angle and one at a 173° scattering angle. The presence of both detectors provides greater flexibility in terms of selecting measurement conditions. Particles can be dispersed in a wide variety of liquids, and only the refractive index and viscosity of the liquid are required to interpret the measurement result. The obtained optical signal exhibits change due to the particles’ relative positions changing randomly, and a graph is generated.

Elamawi et al. [220] reported DLS result of filtered AgNPs produced from *Trichoderma longibrachiatum*; 10 g fungal biomass and 15 g fungal biomass showed three peaks and two peaks with the presence of 39.3, 4.4, 1.5 nm and 41.7 as well as 4.9 nm, respectively. Mohanta et al. [226] reported that DLS resulted from AgNPs synthesized by *Protium serratum* showed 75.56 ± 0.46 nm in size, whereas Guilger-Casagrande et al. [143] reported AgNP-TS and AgNP-T NPs synthesized by *Trichoderma harzianum* representing hydrodynamic diameters of 57.02 ± 1.75 and 81.84 ± 0.67 nm, respectively. NPs synthesized using *Trichoderma harzianum* (AgNP-TS) whereas without enzymatic stimulation (AgNP-T) by the cell wall of *Sclerotinia sclerotiorum*. The AgNPs synthesized by *Enicostemma axillare* leaf extract showed 25 to 80 nm in size and dispersity index (PDI) of 0.412 [227].

#### 2.4.3. X-ray Diffraction (XRD)

X-ray diffraction is one of the most extensive conventional techniques to characterize NPs. XRD is a technique used to determine the crystallographic structure and morphology, which include the crystalline structure, lattice parameters, nature of the phase and crystalline size. The number of constituents influences the increase or decrease in intensity. Changing the atoms in the unit cell causes the changing of diffraction intensity. X-rays are electromagnetic radiation similar to light. However, X-rays have a much shorter wavelength compared to light. They are produced when deceleration of electrically charged particles occurs [228]. The peak positions indicate the translational symmetry shape and size of the particles while the peak intensity indicates the position of atoms located and electron density inside the unit cell. This method is unsuitable for amorphous materials and particles with a size below 3 nm as XRD peaks produced are too broad. Crystalline size is calculated using Scherrer equation [229]. The equation is Full width at half maximum, FWHM (2θ) = bλ/Dcosθ, or D = b2п/FWHM (Q), where 2θ is the scattering angle in radians, λ is the wavelength, Q is the magnitude of the scattering vector, b is a constant, usually a value between 0.89 and 0.94 depending on the function used to fit the peak, and D is the dimension of crystallites. Table 2 shows biogenic AgNPs characterization using XRD.

#### 2.4.4. Transmission Electron Microscopy (TEM)

Transmission electron microscopy (TEM) is used to characterize NPs. Beam of electrons is transmitted through an ultra-thin section of the microscopic object. The beam of electron interacts with the sample and transforms into unscattered electrons, inelastically scattered electrons or elastically scattered electrons. Then, the scattered or unscattered electrons are focused by a series of electromagnetic lenses and projected on the screen. This generates an amplitude-contrast image, a phase-contrast image, electron diffraction or a shadow image with varied darkness depending on density of unscattered electron [237]. TEM provide detailed quantitative chemical information of particles, size distribution and high-resolution images. The ratio of distance between objective lens and the specimen, and objective lens and its image plane is magnification [238].

TEM has a stronger resolution and magnification than scanning electron microscopy (SEM). The role of capping agents and metabolite encapsulation of AgNPs can also be visualize using TEM [239]. The disadvantages of TEM include the need for a large sample section and a high vacuum. Banu et al. [240] reported to use TEM (Japanese JEM-3000 F, Jeol) to study the structural characterization of the AgNPs using drop-coated film and it showed spherical shape and 5 to 50 nm in size. Similarly, AgNPs was characterized using TEM and it revealed spherical shape with the size ranging from 13 to 40 nm [241].

### 2.5. Application of AgNPs in Biomedical Applications

**Figure 9:**
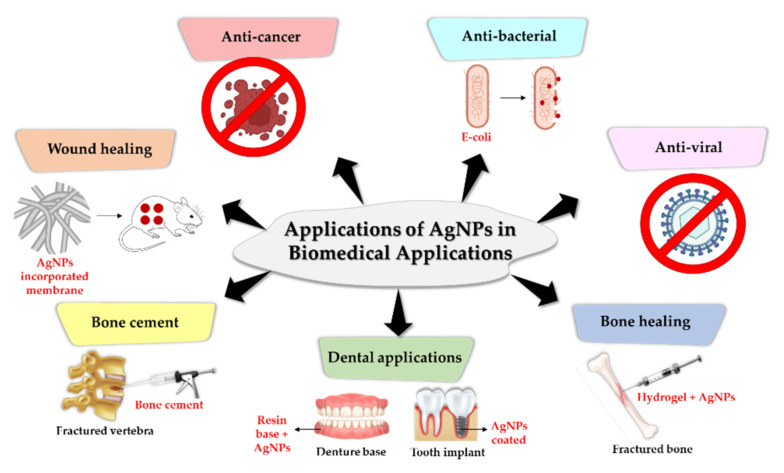
Applications of AgNPs in Biomedical Applications.

The application of AgNPs in biomedical applications plays a crucial role contributing to the development of novel antimicrobial agents, biomaterial and medical device coatings, drug delivery formulations, detection and diagnosis platforms, tissue restoration and regeneration materials and performance-enhanced therapeutic alternatives. The applications of AgNPs in biomedical applications are shown in Figure 9. AgNPs can be a part of antibacterial, antiviral and anticancer therapy. In addition, AgNPs can be incorporated as additives into membrane, bone cement, denture base, tooth implant, fractured bone, catheters and hydrogel to prevent or reduce formation of biofilm or any medical pathogens as well as improve and fasten the recovery of bone growth, wound and gums recovery.

**Figure 10:**
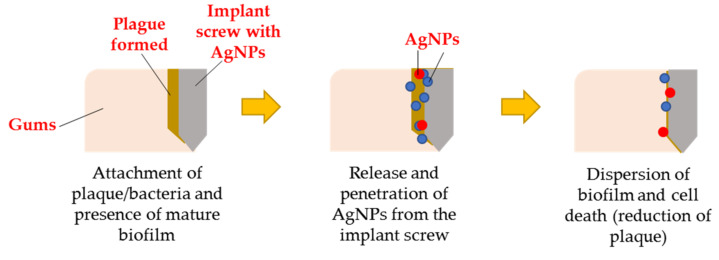
Formation of biofilm and AgNPs application (adapted from [245]).

AgNP applications may be used to combat the alarming and emerging problem of pathogenic drug resistance. Additionally, AgNPs exhibit a broad antibacterial activity against both gram-positive and gram-negative bacteria, which is advantageous for biomedical applications. Both pathogens can adhere to a surface, resulting in the growth of a biofilm. AgNPs were used to penetrate and disperse the biofilm, resulting in the pathogens being released from the infection surface (Figure 10). According to another study, when AgNPs are applied to cells, they penetrate the cell and cause abnormal cell function, structural damage, and ultimately cell death [242]. A study by Neihaya and Zaman [243] reported the changes from black colonies into pink colonies after the application of biosynthesized Ag, which was concluded due the loss of biofilm formation ability. Similarly, another biosynthesized Ag disrupts 80% of Uropathogenic *E. coli* (UPEC) biofilms [244]. As a result, AgNPs could be excellent, dependable, and effective solution for major concerns: antibacterial, antiviral, anticancer therapy, bone healing, bone cement, dental applications and wound healing.

#### 2.5.1. AgNPs for Antibacterial Activities

**Figure 11:**
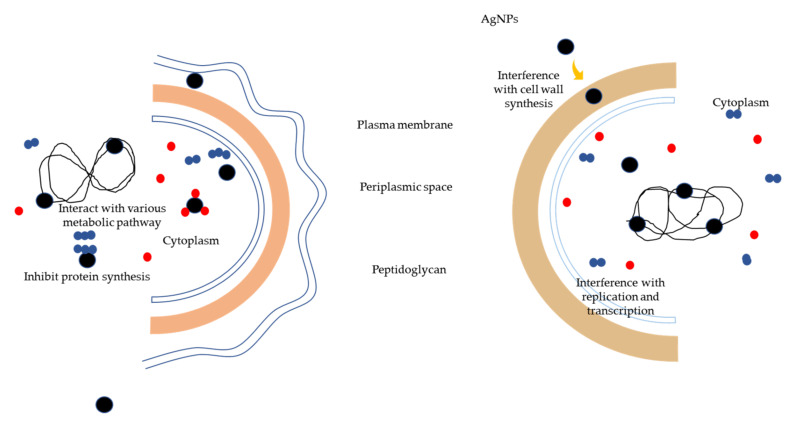
The possible mechanism (ROS activation) towards gram-positive and gram-negative (adapted from Pandey et al. [248]).

Antibiotics have been used to treat antibacterial infections for years; however, due to inconsiderate, misuse, and multiple use of antibiotics, multidrug-resistant (MDR) microorganisms have emerged. Apart from that, many antibiotics have lost their efficacy due to the evolution of resistant strains. As a result, this has posed a serious threat to the global human population, as more than 60% of bacteria that cause nosocomial infections are now resistant to at least one of the most commonly used antibiotics [246]. Multidrug-resistant infections in humans are difficult to treat and result in a lengthy hospital stay [247] *S. enteritidis*, *K. pneumoniae*, *E. coli*, *S. aureus*, and *P. aeruginosa* are just a few of the species that cause infections in biomedical settings. However, the application of AgNPs has emerged as a potential solution to the multidrug resistance problem. AgNPs are a cost-effective and efficient way to combat multidrug-resistant bacteria. Bacteria can be gram-positive or gram-negative. AgNPs can be efficient in inhibiting both gram-positive and gram-negative bacteria. Figure 11 shows the possible mechanism of actions of AgNPs towards gram-negative and gram-positive bacterial cells.

Gram-negative bacteria have a thin peptidoglycan layer with periplasmic membrane and an additional outer membrane, whereas Gram-positive bacteria have a thick peptidoglycan layer with periplasmic membrane. Gram positive bacteria are reported to be more resistant to AgNPs [249,250]. The effect of AgNPs on *E. coli*, *P. aeruginosa*, and *S. aureus* was investigated, and it was concluded that gram-negative strains such as *E. coli* and *P. aeruginosa* are more susceptible to cell wall damage than *S. aureus*, a gram-positive strain with a thick cell wall [251].

Huq and Akter [252] described the synthesis of AgNPs using the supernatant of *Massilia* sp. MAHUQ-52 and their antimicrobial activity against multidrug-resistant pathogens, *Klebsiella pneumoniae* and *Salmonella enteritidis*. These pathogens were tested against AgNPs synthesized in the laboratory and six additional antibiotics. *S. enteritidis* was found to be resistant to five antibiotics tested and to have an inhibition zone of 16.8 ± 0.9 mm for biosynthesized AgNPs. The AgNPs exhibited inhibition zones with a diameter of 17.6 ± 0.5 mm against *K. pneumoniae*, whereas the other five antibiotics exhibited no antibacterial efficacy. The authors concluded that the inhibition is due to AgNPs rupturing the cell wall and causing cell death. Huq [253] investigated the antimicrobial efficacy of AgNPs synthesized by *Pseudo-duganella eburnea* MAHUQ-39.

The antibiotic-resistant human pathogens used were *E. coli*, *S. aureus* and *P. aeruginosa*. MICs of *P. aeruginosa* and *S. aureus* were 6.25 μg/mL and 200 μg/mL and 100 μg/mL whereas MBCs of *P. aeruginosa* and *S. aureus* were 50 μg/mL and 200 μg/mL, respectively. Nanda et al. [254] studied on the biosynthesis of AgNPs using *S. aureus* and their antibacterial activity against human pathogens such as methicillin-resistant *S. epidermis*, methicillin-resistant *S. pyogenes*, *S. typhi* and *K. pneumoniae*. MRSA and MRSE are bacteria found to be resistant to a wide range of broad-spectrum antibiotics. The inhibition zones for MRSE was 18 mm, followed by MRSA was 17.5 mm and *S. pyogenes* was 16 mm. It was also reported that all gram-positive pathogens were inhibited by AgNPs. In another study, Du et al.’s [255] in vitro results of the application of biosynthesized AgNPs using *Novosphingobium* sp. THG-C3 as a potential treatment/prevention against human pathogens, *P. aeruginosa*, *V. cholera*, *S. aureus*, *K. pneumoniae*, *E. coli* and *S. typhi*. The results showed that biosynthesized AgNPs showed an effective antibacterial activity against *S. aureus*, *K. pneumoniae* and *S. typhi* with zone of inhibition of 12, 7 and 7 mm, respectively. There are more literatures available for in-vitro testing against human pathogens (Table 3).

#### 2.5.2. AgNPs for Antiviral Activities

**Figure 12:**
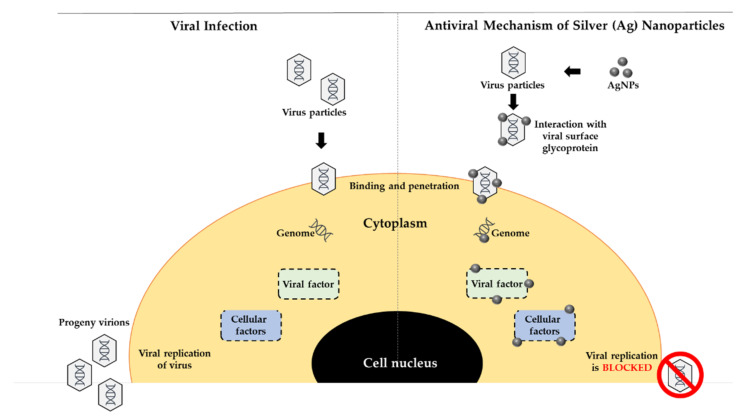
The antiviral activities of AgNPs [273].

There have been recent outbreaks of an infectious disease as a result of the presence of a newly evolving pathogenic virus. Viruses are capable of infecting both eukaryotes and prokaryotes. Virus replication requires living cells, which they damage or even destroy. Systemic infections and severe complications can occur as a result of acute and chronic conditions that develop following the viral infection. Viruses are found in the air, water, and, most commonly, the soil. The newly evolving virus has developed resistance to currently available antiviral drugs, prompting researchers to investigate new drugs and alternative treatment options. As a result, AgNPs have been used as an antiviral agent in the biomedical field. Additionally, biogenic AgNPs are an antiviral agent against HIV-1 [270], herpes virus (HSV-1 and HSV-2) [271], and dengue virus (DEN-2) [272]. The mechanism by which a virus infects a cell is depicted in Figure 12 along with the role of AgNPs as an antiviral agent.

Although there are a few types of research on the effect of AgNPs towards viruses, the details of the interactions are scarce. However, this can be due to the complexity of virus structure that contribute to the limited knowledge of AgNPs mechanism towards the viruses. Salleh et al. [273] stated that there are two ways in which how AgNPs interact with pathogenic virus; (1) Bind to the outer coat of virus and inhibit the attachment of virus towards cell receptors and (2) Bind to the DNA or the RNA of virus and inhibit replication or propagation of the virus. Table 4 shows biosynthesized AgNPs and the antiviral properties towards different types of pathogenic virus.

In addition, Sharma et al. [281] used the medicinal plants *Andrographis paniculate*, *Phyllanthus niruri* and *Tinospora cordifolia* to synthesize AgNPs and tested their antiviral properties against chikungunya virus. Based on DLS, the average sizes of AgNPs synthesized *by A. paniculate*, *P. niruri* and *T. cordifolia* produced was 68.06, 28.38 and 37.10 nm, respectively. It was reported that *A. paniculate* AgNPs exhibited the highest antiviral properties against chikungunya virus. In another study, Sreekanth et al. [282] demonstrated the biosynthesis of AgNPs using extract of *Panax ginseng* roots and tested against influenza virus. The AgNPs produced are spherical and 5 to 15 nm in size. It was reported that only slight anti-influenza was detected at concentrations of 0.005, 0.01 and 0.15 M and increased at concentration of 0.02 and 0.25 M of AgNPs.

#### 2.5.3. AgNPs for Anticancer Therapy

Cancer and its secondary complications account for an alarming number of deaths worldwide, owing to insufficient efficacy, severe side effects from currently available chemotherapeutic agents, and poor prognosis. Breast cancer (13.7%) is the most frequently reported type of cancer worldwide, followed by colorectal cancer with 11% [283]. Secondary complications associated with chemotherapies used to treat these two leading cancers include infection with bacteria, viruses, and fungi, as well as a compromised immune system. The conventional treatment of cancer, which includes surgery, radiation therapy, and chemotherapy, results in ineffective anticancer therapy, owing to the treatment’s decreased selectivity and specificity [284]. Additionally, cytotoxic drugs used to treat breast cancer, such as doxorubin, cis-plation, and bleomycin, have drawbacks and are ineffective [285]. As a result, nanoparticles such as AgNPs have the potential to act as anti-cancer nanomedicines (Table 5).

Nguyen and colleagues [286] reported on the treatment of hepatocellular carcinoma (HepG2) and breast cancer (MCF-7) with AgNPs synthesized in the presence of *Ganoderma lucidum* (GL). Additionally, they demonstrated that spherical nanoparticles of AgNPs/GL with a physical size of 10.72 nm effectively inhibited the proliferation of HepG2 and MCF-7 cells. In another study, Narasimha et al. [287] synthesized AgNPs from *Eucalyptus globulus* L. leaf extract and used them to treat colorectal cancer cell lines (HCT116). AgNPs were produced with a physical size of 20 nm and a spherical shape. The AgNPs arrested the cell cycle, increased the expression of apoptotic genes, and decreased the expression of antiapoptotic, inflammatory, and stem cell markers in HCT116 cells.

In another similar study, the *Acalypha indica* Linn AgNPs inhibits 40% of human breast cancer cells (MDA-MB-231) [288]. Deeb et al. [289] reported the in vivo and in vitro studies using AgNPs biosynthesized with *Arthrospira platenses*, *Microcystis aeruginosa* and *Chlorella vulgarisactive* metabolites against breast cancer. Results showed the safety usage of bio-AgNPs was 0.1 mg/mL on PBMCs cells and 1.5 mg/mL on the tested mice. The biogenic AgNPs showed dose-dependent cytotoxicity against HepG-2, CaCo-2 and MCF-7 cell lines. The *Arthrospira* bio-AgNPs displayed a delay in tumour growth and prolonged survival in mice model, which explained that the treatment induced cellular apoptosis in cancer cell by down regulating of surviving, MMP7, TGF and Bcl2 genes expressions. Gajendran et al. [290] reported the inhibition of 50% of proliferation of human breast cell line (MCF7) after 24 h incubation using 20 μg/mL AgNPs produced by *Danura inoxia.* The AgNPs can result in cell membrane integrity, apoptosis, oxidative stress and reduced DNA synthesis. The AgNPs (5 μg/mL) biosynthesized by *Dendrophthoe falcata* were spherical and 5 to 45 nm in size and caused MCF-7 to lose their 50% viability [291].

**Table 5:** The anticancer results of green synthesized AgNPs.

Organism	Source of AgNPs	Cancer Cell Line	IC50 Value	Size and Shape of AgNPs	References
Mixture of *Curcuma longa* and *Zingiber officinale*	Plant	HT-29	150 μg/mL	Spherical; 20–51 nm	[292]
*Solanum trilobatum*	Plant	MCF-7	30 μg/mL	Spherical; 12.5–41.9 nm	[293]
*Aspergillus terreus ITCC 9932*	Fungus	MCF-7	25.24 ± 0.990 μg/mL	Spherical; 25 nm	[294]
*Aspergillus niger* *Aspergillus michelle* *Aspergillus japonicus*	Fungus	MCF-7	2.46 μg/mL 3.12 μg/mL 1.47 μg/mL	Varying in sizes Varying in sizes ~100 nm	[295]
*Agaricus bisporus*	Fungus	MCF-7	50 μg/mL	Spherical; 8–20 nm	[296]
*Endophytic bacterium*	Bacteria	MCF-7	50 μg/mL	Spherical; 83–176 nm	[297]
*Dimocarpus longan*	Plant	PC3	10 μg/mL	Cubical; 9–32 nm	[298]
*Ginkgo biloba*	Plant	HeLA, SiHa	Dose dependent	Spherical; 40 ± 1.2 nm	[299]
*Punica granatum*	Plant	A5449	5 μg/mL	Spherical; 6–45 nm	[300]
*Detarium microcarpum*	Plant	HeLa, PANC-1	31.5 μg/mL, 84 μg/mL	Spherical, circular, rectangular; 62–103 nm (TEM)	[301]
*Punica granatum*	Plant	HeLa	100 μg/mL	46.1 nm	[302]
*Fagonia indica*	Plant	MCF-7	12.35 μg/mL	Spherical;10–60 nm	[303]
*Alternanthera sessilis*	Plant	PC3	6.85 μg/mL	Spherical; 30–50 nm	[304]
*Oscillatoria limnetica*	Bacteria	MCF-7, HCT-116	6.147 μg/mL, 5.369 μg/mL	Quasi-spherical; 3.30–17.97 nm	[119]
*Rhizopus stolonifer*	Fungus	EAC, HT-29	2.15 μg/mL, 2 μg/mL	Spherical; 5–50 nm	[240]

Kanipandian et al. [241] performed a study in which they used biosynthesized AgNPs with a size range of 13–40 nm to induce mitochondrial-dependent apoptosis in lung adenocarcinoma. Cell cycle arrest was induced by the spherical AgNPs. Ferreira et al. [305] conducted in vitro and in vivo studies with biogenic AgNPs produced by *Fusarium* sp. In vitro treatment with IC50 decreased clonogenic survival, whereas treatment with IC25 had a negligible effect on clonogenic survival.

#### 2.5.4. AgNPs for Bone Healing

Bone is an active tissue with self-repairing capability. However, this capability is usually compromised when infection occurs in bone defects. Large defects resulted from tumour resection, genetic malformation or severe trauma can be replaced or restored by implanting bone grafts [306]. Implant failure is mainly associated with infection and often causes financial burden, patient suffering and even fatalities [307,308]. In addition, it was also reported that implant related infection can result in amputation in patients [309].

Around 50 to 60% of the infections commonly caused by *Staphylococcus* spp. [310]. These bacteria are commonly treated with application of antibiotic, but as time goes by, these organisms gained resistant towards antibiotics used. In addition, they have ability to rapidly form biofilm, within which they are protected from antibiotics including, the natural antibiotics [311]. In this application, AgNPs are used due to their antimicrobial effect in tumour prostheses, trauma implant, combined with hydroxyapatite coatings and bone cement [312].

Orthopaedic infections are typically responsible for bone destruction and implant loosening [313]. AgNPs incorporated into crystallised hydroxyapatite (HA) or titanium scaffolds demonstrated significant antibacterial activity against both bacteria [314]. Felix and Muthu [315] discovered that AgNPs impregnated with bioscaffolds improve bone healing.

#### 2.5.5. AgNPs for Bone Cement

Bone cement is used in orthopaedic surgery to secure devices in dental and arthroplasty sites, as well as to join fractured bones together. The surgery involved the replacement of a damaged body part with an artificial one due to an acute injury or degenerative disease. The most frequently performed operations were disc replacements in the knee, hip, and spine [316]. According to data from the National Healthcare Safety Network, infections occurred in up to 2.3% joint replacement surgeries in the United States and with the highest infection rate occurring in 15% of ankle replacement surgeries. Antibiotics were traditionally administered orally or released from bone cement to prevent infections [317]. However, antibiotic use has resulted in the development of antibiotic-resistant bacteria. As a result, a novel approach was required, and thus the development of a non-antibiotic technique occurred [316]. Maybe AgNPs were incorporated into bone cement for antibacterial purposes without impairing the material’s cytotoxicity [318,319].

#### 2.5.6. AgNPs for Dental Applications

Plaque formation is a contributing factor to the development of dental diseases. The oral cavity is home to a variety of microorganisms. Polymicrobial communities grow and form biofilms in the oral cavity. These biofilms are capable of causing a variety of local diseases, including peri-implant and periodontal diseases, which can result in implant failure or tooth loss [320]. Peri-implants are associated with severe complications following implant replacement [321]. Peri-implantitis mucositis occurred in up to 50% of cases and peri-implantitis occurred in up to 43% [322]. Lang et al. [323] reported that there is no difference in the formation of bacterial biofilms on tooth and implant surfaces, but that surface roughness may have an effect. The polymicrobial communities includes *Streptococcus sanguis*, *Streptococcus oralis*, *Streptococcus mitis*, *Eikenella corrodens* and *Veillonella atypica*.

Some dental biomaterials were incorporated with AgNPs to reduce biofilm formation. Thomas et al. [324] biosynthesized AgNPs using *Bacillus amyloliquefaciens* SJ14 culture and extract of *Curcuma aromatica rhizome* and incorporated in Polymethyl methacrylate (PMMA), showed inhibition against cariogenic bacteria, *S. mutans* from colonizing dental restorative material. The biosynthesized AgNPs incorporated PMMA can be applied as a dental material. Modifications of PMMA surface by incorporating an antibacterial agent is to avoid or minimize microbial attachment and their posterior colonization [325]. The AgNPs were biosynthesized using *Geranium maculatum* leaves. AgNPs is more effective against pathogens like *Candida albicans* [326].

In preventive dentistry, AgNPs were used to infiltrate carious lesions and precipitate, leading to enamel hardening. Al-Nerabieah et al. [327] reported in vivo studies using Nano-silver Fluoride with green tea extract (NSF-GTE) to arrest cavitated lesions and teeth in preschool children was used as subject. The study recorded 67.4% effective in arresting dentin carious lesions in both posterior and anterior primary teeth for six months. Mineral trioxide aggregate (MTA) is one of the materials of choice for the repair of root perforations and was introduced by Torabinejad et al. [328]. Many studies have reported MTA was mixed with the various additives. In a study done by Bahador et al. [329], in vitro result of the application of AgNPs as a potential mixture of mineral trioxide aggregate (MTA) to prevent the infection of *Porphyromonas gingivalis*. Results showed that IMTA containing 12% and 25% completely inhibited the proliferation of *P. gingivalis* in a dose dependent manner in root perforations. Another study reported the application AgNPs biosynthesized by aqueous leaf extract of *Mangifera indica* in dental restoration applications [330]. The AgNPs reinformed glass ionomer cement (GIC) enhanced the mechanical strength of the conventional dental implant materials and act as a surface coating to act against *S. aureus* and *E. coli*. Paul et al. [331] investigated the use of AgNPs synthesized by white pepper oleoresin in testing against oral pathogens such as *S. aureus*, *S. mutans* and *Pseudomonas* sp. Results reported that biosynthesized AgNPs inhibited all three oral pathogens. Similarly, Umai et al. [332] reported the testing of AgNPs biosynthesized by *Olea europaea* and tested against major oral pathogens such as *C. albicans* and *S. mutans*. The AgNPs inhibited the growth and biofilm formation of *S. mutans* and *C. albicans*. The biosynthesized AgNPs can be beneficial as a surface coating of conventional dental implants to prevent infections. In another, Halkai et al. [333] suggested that AgNPs synthesized by fungus *Fusarium semitectum* showed effective antibacterial activity against endo-perio pathogens such as *P. gingivalis*, *B. pumilus* and *E. faecalis*. The biosynthesized AgNPs has the potential to be a solution in the management of endodontic, periodontal, and combined lesion.

#### 2.5.7. AgNPs for Catheters

Central venous catheters (CVCs) are used for a variety of purposes, including monitoring hemodynamic, administering medications, providing nutritional support, administering intravenous fluids, and administering blood products [334]. Forssman developed one of the earliest techniques for CVC in 1929 and was awarded the 1956 Nobel Prize in Medicine for his work in this field [335]. However, these catheters have the potential to introduce infection into the bloodstream [336]. In clinics, the morbidity and mortality rates associated with catheter-related bloodstream infection (CRBSI) increased [337]. Infection occurs when bacteria attach to the surface of CVCs and grow to form biofilms under favourable conditions [338]. According to a research study, CRBSI are caused by a variety of *Staphylococcus aureus* strains, with 82 percent of them being methicillin resistant strains capable of forming biofilms and bacterial dispersion [339]. Hence, many approaches have been administrated to reduce CRBSI. The Institute for Healthcare Improvement had recommended a promising approach, the use of central line bundles. These bundles consist of five different components of care, which include optimal catheter site selection, hand hygiene, maximal barrier precautions, chlorhexidine skin antisepsis and daily review of central line necessity [340]. However, alternative method such as impregnating or coating catheters with AgNPs internally and externally also was introduced. Recent studies had reported AgNPs-modified catheters as nontoxic medical equipment with inhibitory effects against infection related issues by releasing AgNPs [338,341,342].

LewisOscar et al. [343] demonstrated in vitro study of AgNPs synthesized by *Spirulina platensis* tested against biofilm formation of *P. aeruginosa*. The authors reported that 100 μg/mL showed the maximum inhibition, 85.63% of biofilm formation and can be a potential coating medical device such as urinary catheter. Similarly, Rahuman et al. [344] studied the inhibition of urinary tract infection pathogen using biosynthesized silver coated urinary catheter. The AgNPs coated urinary catheter showed excellent antibacterial activity towards *S. aureus* and *P. aeruginosa* as well as the highest biofilm inhibition, with 85.8% against *P. aeruginosa*.

#### 2.5.8. AgNPs for Wound Healing

Wounds develop as a result of skin tissues being cut, punctured, torn, or burned in response to stimuli or trauma. There are two types of wounds: acute and chronic, which refer to the length of time required for healing and other complications [345]. Wound healing necessitates extensive communication between the extracellular matrix and the skin’s various cellular constituents. Additionally, wound healing requires the involvement of a variety of factors, including coagulation factors, connective tissue, cytokines, the vascular system, growth factors, and cell types. Failure to treat the wound properly will result in a chronic wound. As a result, nanotechnology-based approaches to wound therapy were developed. Nanomaterials can be used as a drug delivery vehicle for wound healing or as a delivery agent for wound repair [346].

It has been demonstrated that *Fusarium oxysporum* biosynthesis of AgNP is accurate in vivo. The produced AgNPs have a diameter of between 20 and 40 nm and are then complexed with Enox for 28 days of treatment in an in vivo burn wound model. Both AgNP and AgNP-Enox demonstrated wound healing rates of between 89 and 95%. The AgNP-Enox group had an increase in urea levels, indicating an increase in proteolysis due to inflammation. There was no evidence of bleeding or toxic effects observed in terms of haemoglobin and biochemical parameters. Krishnan et al. [347] synthesized biosynthetic biogenic cubical from *Brevibacillus brevis* KN8(2) culture filtrate and evaluated it in vivo on diabetic mice for early wound healing. The biogenic cubical AgNPs inhibited MMP-2 and MMP-9 mRNA and protein expression in wounded granulation tissues, resulting in rapid wound healing. When compared to the control group, mice treated with AgNPs demonstrated a rapid decrease in wound area, complete epithelialization, and a significant increase in fibroblast and collagen deposition (no AgNPs). When the plant extract of *Peltophorum Pterocarpum* was tested for wound healing activity against fibroblast 3T3, it produced AgNPs with a diameter of 20 to 50 nm and a spherical shape. Following 48 h of exposure to AgNPs loaded with hydrogel, increased cell migration in fibroblast cells following the scratch, there was a noticeable increase in the number of fibroblasts, suggesting that they migrated or proliferated. When fibroblasts differentiate into myo-fibroblasts, contractile elements are expressed. Furthermore, it promotes wound healing. Using an aqueous leaf extract of *Ardisia solanacea*, we synthesized AgNPs with a diameter of 21 nm. We performed in vitro wound healing assays using normal fibroblast cell lines, BJ-5Ta, and observed the healing process using phase contrast microscopy [348]. The wound healing activity of AgNPs was found to be beneficial to the study’s results.

Garg et al. [349] demonstrated the therapeutic potential of biogenic AgNPs synthesized from *Arnebia nobilis* root extract hydrogel. The healing capacity of AgNPs with a diameter of 40 to 70 nm and a spherical shape was investigated using an excision wound model. The hydrogel formulation significantly improved wound contraction and closure during the first and second weeks. After 14 days, the albino rats healed wounds 9.34% faster than the control group, but the control group healed wounds 1.78% faster than the albino group after 21 days. In another study, Dhapte et al. [350] used biogenic AgNPs derived from aqueous *Bryonia laciniosa* leaves extract to conduct in vitro and in vivo experiments. AgNPs synthesized at a size of 153 nm appeared to possess antibacterial activity against gram-negative and gram-positive bacteria. According to research conducted on rats, the control group experienced a 47.0–72.19% reduction in the size of the wound, whereas the commercially available preparation of “Silver Sulfadiazine” healed 78.1–31.35% of the wounds. Additionally, 92.54 ± 3.67% of the wound was treated with AgNPs gel. AgNP gel-treated mice demonstrated rapid wound epithelialization, indicating that it may be used in biomedical wound healing. Similarly, biogenic silver nanoparticle-based gel was prepared in gellan gum and demonstrated approximately 98% wound healing [351].

## 3. Conclusions

AgNPs can be synthesized using various method and biological synthesis is the most preferred method in producing AgNPs used in biomedical fields. Biomedicine is a branch of medical science that related to development of diagnostic modalities, treatment for current and emerging treat to human. Biomedical pathogenic organism is an organism that can cause disease in a host (person) that can bring disease or sickness to host (humankind) [352]. The most common methods of AgNPs characterization are UV-Vis, FTIR, DLS, XRD and SEM analysis. The applications of AgNPs in biomedical field increase by years as they are efficient and effective against biomedical pathogens. The application of nanotechnology, specifically AgNPs, could be one of the best solutions in disease control, infection control, anticancer therapy, dental applications, coating system and wound healing and many more biomedical applications with minimal or no side effects.

## Figures and Tables

**Table 1:** Reported size and shape of AgNPs synthesized by microorganisms.

Name of Bacteria	Size (nm)	Shape	References
*Bacillus cereus*	10–30	spherical	[112]
*Nocardiopsis* sp. MBRC-1	45 ± 0.15	spherical	[113]
*Stenotrophomonas*	40–60	multi-shaped	[114]
*Acinetobacter calcoaceticus*	8–12	spherical	[115]
*Escherichia coli*	20–50	spherical	[116]
*Leuconostoc lactis*	35	spherical	[117]
*Haemophilus influenzae*	80–101	spherical	[118]
Cyanobacterium *Oscillatoria limnetica*	3.30–17.97	quasi-spherical	[119]
Cyanobacteria *De sertifilum* sp.	5–26	spherical	[120]
*Sphingobium* sp. MAH 11	7–22	spherical	[121]
*Bacillus licheniformis*	7–22	spherical	[122]
*Klebsiella pneumonia*	26.84–44.42	spherical	[123]
*Pseudomonas stutzeri* AG259	200	Triangle, hexagon and spherical	[124]
*Proteus mirabilis* PTCC 1710	10–20	spherical	[125]
*Lysinibacillus xylanilyticus* strain MAHUQ-40	8–30	spherical	[126]
*Bacillus* sp. AZ1	7–31	spherical	[127]
*Streptomyces* sp. 09 PBT 005	198–595	spherical	[128]
*Exiguobacterium sp. KNU1*	4.4	spherical	[129]
*Shewanella* sp. ARY1	38	spherical	[130]
*Lactobacillus* sp. strain LCM5	13.84 ± 4.56	spherical	[131]
*Bacillus* sp-*Brevibacillus borstelensis_MTCC10642*	5–15	cubical	[132]

**Table 2:** Biogenic AgNPs characterization with XRD.

Plants/Bacteria/Algae	Average Crystallite Size (nm)	AgNPs Peaks at 2θ Angles	References
*Nocardiopsis* sp. MBRC-1	45 ± 0.15 nm	38.44°, 44.38°, 56.77°, 64.38° and 77.50°	[113]
*Urtica dioica* (Linn.) leaf extract	20–30 nm	38.45°, 46.35°, 64.75° and 78.05°	[219]
*Wedelia urticifolia* (Blume) DC	179.3 nm, 90.38 nm and 80.28 nm	38.22°, 44.42°, 64.56° and 77.50°	[230]
*Allophylus serratus* Leaf and Leaf Derived *Callus* Extracts	42 nm (leaf) and 44 nm (extract)	32.5°, 38.3°, 44.4°, 64.6°, and 76.8° and 32.4°, 38.3°, 44.5°, 64.5°, and 76.7°	[231]
*Erythrina indica* leaf extract	~28.19 nm	24.934°, 37.0359° and 43.8572°	[232]
*Caesalpinia ferrea* seed extract	30–50 nm	38.15°, 44.25°, 64.47°, 77.38° and 81.64°	[233]
*Pandanus odorifer* leaf extract	10–50 nm	37.2°, 43.4°, 63.5° and 76.6°	[234]
*M. azedarach* leaf extract	21 nm	37.88°, 44.31°, 64.34° and 77.39°	[235]
Brown seaweed *Colpomenia sinuosa*	54–85 nm	11.58°, 32.04°, 37.89° and 46.96°	[236]

**Table 3:** In-vitro testing against human pathogens.

Organism	Source	Size and Shape	Test Organism	References
*Origanum heracleoticum* L.	Bacteria	Spherical; 30–40 nm	*E. coli, P. aeruginosa, S. aureus, S. pneumoniae, K. pneumoniae*	[256]
*Sporosarcina koreensis* DC4	Bacteria	Spherical; 102 nm	*V. parahaemolyticus*, *E. coli*, *S. enterica*, *B. anthracis*, *B. cereus* and *S. aureus*	[257]
*Dodonaea viscosa* extract	Plant	Spherical; 16 nm	*E. coli*, *K. pneumoniae*, *P. fluorescens*, *S. aureus*, *B. subtilis*	[258]
Marine *Pseudomonas* sp. H64	Bacteria	Spherical; 3–22 nm	*B. subtilis*, *S. faecalis*, *S. aureus*, *E. coli*, *A. hydrophila*, *V. parahaemolyticus*	[259]
*Aloe arborescens*	Plant	Spherical; 38 ± 2 nm	*P. aeruginosa*, *S. aureus*	[260]
*Acorus calamus*	Plant	Spherical; 20–35 nm	*S. aureus*, *E. coli*	[261]
*Fusarium scirpi*	Fungus	Quasi-spherical; 2–20 nm	*E. coli*	[244]
*Artemisia marschalliana* extract	Plant	Spherical; 5–50 nm	*S. aureus, B. cereus, A. baumannii*	[262]
*Moringa oleifera*	Plant	Spherical; 8 nm	*K. pneumoniae*, *S. aureus*	[263]
*Caulerpa racemose*	Algae	Spherical and few triangular; 5–25 nm	*S. aureus, P. mirabilis*	[264]
*Leptolyngbya* strain JSC-1	Algae	Spherical; 5–50 nm	*S. aureus, E. coli*	[265]
*Penicillium oxalicum* strain LA-1	Fungus	Spherical; 52.26 nm	*K. pneumoniae*, *V. cholerae*, *E. coli*, *M. luteus*, *M. smegmatis*, *B. subtilis*	[266]
*Aspergillus niger*	Fungus	Spherical; 1–10 nm	*C. krusei*, *C. parapsilosis*, *C. tropicalis*, *A. flavus*, *A. fumigates*, *S. aureus*, *P. aeruginosa*, *E. coli*	[267]
Alga *Syridia fusiformis*	Algae	Spherical, Triangle, Pseudo-spherical, rectangle; 5–50 nm	*K. pneumoniae, S. aureus*	[268]
*Ipomoea asarifolia*	Plant	Spherical; 20–60 nm	*B. subtilis*, *S. aureus*, *E. coli*, *K. pneumoniae*	[269]

**Table 4:** The antiviral properties of biogenic AgNPs.

Virus	Family	Source of AgNPs	Size of AgNPs	Mechanism	References
Herpes simplex virus type 1 and type 2 (HSV-1 & HSV-2)	*Hepesviridae*	*Dryopteris species*, *Musa paradisiaca*, *Catharanthus roseus*, *Selaginella bryopteris*, *Syzygium cumini*	4–31 nm	Block interaction of viral cells	[274]
Human parainfluenza virus type 3 (HPIV3)	*Paramyxoviridae*				
Zika virus	*Flaviviridae*	*Rhazya stricta*	20–40 nm	Penetrate the infectious agent	[275]
Dengue virus (DEN-2)	*Flaviviridae*	*Bruguiera cylindrica*	30–70 nm	Inhibitory effect on viral RNA synthesis	[276]
		*Carica papaya* leaf	10–35 nm	Inhibition of viral replication	[277]
		*Leucas aspera* and *Hyptis suaveolens*	7–22 nm	Capping facilitated surface activity makes these AgNPs a tool for vector control	[278]
HSV-1, HAV-10 and CoxB4 virus	*Herpesviridae*	*Lampranthus coccineus*	10.12–27.89 nm	Interact with herpes simplex thymidine kinase, hepatitis A 3c proteinase and Coxsackie virus B4 3c protease	[279]
		*Malephora lutea F. Aizoaceae*	8.91–14.48 nm	Interact with herpes simplex thymidine kinase, hepatitis A 3c proteinase and Coxsackie virus B4 3c protease	[279]
Respiratory Syncytial Virus (RSV)	*Pneumoviridae*	*Curcuma longa*	0.23 nm	Prevent the virus from entering cells and inhibition of viral replication	[280]
HIV-1	*Retroviridae*	*Rhizophora lamaeckii*	12–28 nm	HIV-1 reverse transcriptase inhibitory activity	[270]
Herpes Simplex Virus (HSV-I,II)	*Herpesviridae*	*Sargassum withtii*	-	Prevent the virus from entering cells	[271]

## Data Availability

Not applicable.
